# Supplementary material for: What are the determinants of variation in caretaker satisfaction with sick child consultations? A cross-sectional analysis in five low-income and middle-income countries
Source: BMJ Open. 2023 Dec 19;13(12):e071037. doi: 10.1136/bmjopen-2022-071037 (PMC10749010; doi:10.1136/bmjopen-2022-071037)
Supplement: Supplementary data [file bmjopen-2022-071037supp001.pdf]

Supplementary Files

Box 1. Explained proportion of variance

We applied Snijders and Bosker's method to measure the explained proportion of variance in the logistic multilevel model. We assumed that the dichotomous outcome Y could be conceived as generated through a threshold model with underlying variable $\check{Y}$, denoted as:

$$\check{Y}_{ij} = \gamma^0 + \sum_{h=1}^{\gamma} \gamma^h X_{hji} + U_{0j} + R_{ij}$$

Hence the total variance of Y_{ij} was equal to:

$$\text{Var}\ (Y_{ij}) = \sigma_F^2 + \tau_0^2 + \sigma_R^2$$

where σ_F^2 is the explained variance, which is the observed variance of the linear predictor $\hat{Y}_{ij} = \gamma^0 + \sum_{h=1}^{\gamma} \gamma^h X_{hji}$, and τ_0^2 and σ_R^2 are unexplained variances at levels 2 and 1, which were var (U_{0j}) and var (R_{ij}), respectively. The proportion of total variance explained by the fixed effects in the model, corresponding to an R^2 equivalent for multilevel logistic regression, is defined by:

$$R_{dicho}^2 = \frac{\sigma_F^2}{\sigma_F^2 + \tau_0^2 + \sigma_R^2}$$

When considering only the unexplained part of the total variance, the proportion attributed to the health facilities level (called residual intraclass correlation) was evaluated as:

$$\rho_I = \frac{\tau_0^2}{\tau_0^2 + \sigma_R^2}$$

Table S1. Items included in the service readiness indicator for preventive and curative child health services

	Afghanistan (N = 556)	DRC (N = 2592)	Haiti (N = 2093)	Malawi (N = 3177)	Tanzania (N = 4731)	Total (N = 13149)
Staff and training						
Guidelines for IMCI	90 (16.2%)	1408 (54.3%)	483 (23.1%)	1364 (42.9%)	3006 (63.5%)	6351 (48.3%)
Guidelines for growth monitoring	86 (27.5%)	1057 (58.9%)	599 (44.9%)	1089 (38.5%)	1444 (32.8%)	4275 (40.1%)
Staff trained in IMCI	113 (20.3%)	1575 (60.8%)	716 (34.2%)	1041 (32.8%)	1430 (30.2%)	4875 (37.1%)
Equipment						
Child and infant scale	505 (90.8%)	2347 (90.5%)	1775 (84.8%)	2714 (85.4%)	3085 (65.2%)	10426 (79.3%)
Length/height measuring equipment	154 (49.2%)	1274 (70.9%)	1162 (87.2%)	2546 (90.0%)	1865 (42.4%)	7001 (65.6%)
Thermometer	420 (75.5%)	2482 (95.8%)	1975 (94.4%)	2741 (86.3%)	3991 (84.4%)	11609 (88.3%)
Stethoscope	551 (99.1%)	2426 (93.6%)	2051 (98.0%)	2932 (92.3%)	4374 (92.5%)	12334 (93.8%)
Growth chart	123 (39.3%)	1592 (88.6%)	691 (51.8%)	1785 (63.1%)	3191 (72.5%)	7382 (69.2%)
Diagnostics						
Haemoglobin / anemia testing	389 (70.0%)	2039 (83.3%)	332 (19.9%)	806 (28.6%)	2348 (65.1%)	5914 (53.3%)
Test for parasite in stool (microscopy)	446 (80.2%)	1895 (77.4%)	1456 (87.1%)	773 (27.5%)	2236 (62.0%)	6806 (61.3%)
Malaria testing	464 (83.5%)	2316 (94.6%)	1600 (95.7%)	2522 (89.6%)	3286 (91.1%)	10188 (91.8%)
Medicines and commodities						
Oral rehydration	496 (89.2%)	2092 (81.5%)	1427 (68.2%)	2899 (91.5%)	4021 (85.2%)	10935 (83.4%)
Amoxicillin syrup or tablets	528 (95.0%)	2167 (84.4%)	1973 (94.3%)	2949 (93.1%)	4197 (88.9%)	11814 (90.2%)
Co-trimoxazole syrup or suspension	442 (79.5%)	1339 (52.2%)	1560 (74.5%)	2379 (75.1%)	3683 (78.0%)	9403 (71.8%)
Paracetamol syrup or suspension	493 (88.7%)	1188 (46.3%)	1720 (82.2%)	2304 (72.7%)	3134 (66.4%)	8839 (67.5%)
Vitamin A capsule	470 (84.5%)	1170 (45.6%)	1258 (60.1%)	1477 (46.6%)	3119 (66.1%)	7494 (57.2%)
Albendazole or mebendazole	488 (87.8%)	2174 (84.7%)	1861 (88.9%)	3062 (96.7%)	4227 (89.6%)	11812 (90.1%)
Zinc tablet or syrup	409 (73.6%)	1729 (67.4%)	919 (43.9%)	2722 (85.9%)	2578 (54.6%)	8357 (63.8%)

Table S2. Items included in the technical quality indicator for children older than 2 months

	**Afghanistan**	**DRC**	**Haiti**	**Malawi**	**Tanzania**	**Total**
	(N = 516)	(N = 2452)	(N = 1799)	(N = 3029)	(N = 4516)	(N = 12312)
Provider asked / caretaker mentioned if child unable to drink or breastfeed	70 (13.8%)	526 (21.0%)	319 (18.1%)	848 (27.7%)	1348 (29.7%)	3111 (25.1%)
Provider asked / caretaker mentioned cough or difficult breathing	337 (66.5%)	1576 (63.0%)	1223 (69.4%)	2206 (72.0%)	3498 (77.0%)	8840 (71.4%)
Provider asked / caretaker mentioned diarrhea	203 (40.0%)	1105 (44.1%)	648 (36.8%)	1273 (41.5%)	2813 (62.0%)	6042 (48.8%)
Provider asked / caretaker mentioned fever	384 (75.7%)	2251 (89.9%)	1274 (72.3%)	2465 (80.4%)	4216 (92.9%)	10590 (85.6%)
Provider asked / caretaker mentioned vomiting	180 (35.5%)	1084 (43.3%)	486 (27.6%)	1134 (37.0%)	2352 (51.8%)	5236 (42.3%)
Provider asked / caretaker mentioned convulsions	32 (6.3%)	259 (10.3%)	69 (3.9%)	285 (9.3%)	1041 (22.9%)	1686 (13.6%)
Provider asked about mother's HIV status	0 (0.0%)	36 (1.4%)	36 (2.0%)	216 (7.0%)	377 (8.3%)	665 (5.4%)
Provider asked / caretaker mentioned ear pain	54 (10.7%)	130 (5.2%)	102 (5.8%)	161 (5.3%)	767 (16.9%)	1214 (9.8%)
Provider counted respiration for 60 seconds	79 (15.6%)	691 (27.6%)	366 (20.8%)	501 (16.3%)	644 (14.2%)	2281 (18.4%)
Provider weighed client	240 (47.3%)	1903 (76.0%)	1446 (82.1%)	638 (20.8%)	673 (14.8%)	4900 (39.6%)
Provider plotted weight on growth chart	28 (5.5%)	154 (6.2%)	178 (10.1%)	179 (5.8%)	190 (4.2%)	729 (5.9%)
Provider took temperature	308 (60.7%)	2334 (93.2%)	1562 (88.7%)	2131 (69.5%)	2801 (61.7%)	9136 (73.8%)
Provider checked palms / conjunctiva / mouth for pallor	112 (22.1%)	1911 (76.3%)	909 (51.6%)	1429 (46.6%)	2052 (45.2%)	6413 (51.8%)
Provider checked for oedema	27 (5.3%)	235 (9.4%)	101 (5.7%)	266 (8.7%)	334 (7.4%)	963 (7.8%)
Provider checked vaccination card or vaccinated	54 (10.7%)	551 (22.0%)	475 (27.0%)	2412 (78.7%)	1716 (37.8%)	5208 (42.1%)
Provider asked if child received vitamin A within past 6months	4 (0.8%)	146 (5.8%)	41 (2.3%)	71 (2.3%)	174 (3.8%)	436 (3.5%)
Provider asked if child received any deworming medication in past 6 months	5 (1.0%)	303 (12.1%)	54 (3.1%)	69 (2.3%)	266 (5.9%)	697 (5.6%)
Provider explained dosing if medication prescribed	225 (45.6%)	1220 (49.9%)	1043 (65.8%)	1597 (52.6%)	1686 (38.0%)	5771 (48.1%)
Provider recommended food / liquid intake	174 (34.3%)	680 (27.2%)	694 (39.4%)	581 (18.9%)	1190 (26.2%)	3319 (26.8%)
Provider described ≥ 1 danger sign requiring return to facility	44 (8.7%)	289 (11.5%)	90 (5.1%)	379 (12.4%)	770 (17.0%)	1572 (12.7%)
Provider discussed follow-up appointment	26 (5.1%)	340 (13.6%)	628 (35.7%)	744 (24.3%)	1016 (22.4%)	2754 (22.3%)
Provider stated diagnosis to caretaker	178 (35.1%)	846 (33.8%)	266 (15.1%)	1365 (44.5%)	2205 (48.6%)	4860 (39.3%)

Table S3. Items included in the technical quality indicator for children 2 months or younger

	Afghanistan	DRC	Haiti	Malawi	Tanzania	Total
	(N = 49)	(N = 87)	(N = 341)	(N = 109)	(N = 191)	(N = 777)
Provider asked / caretaker mentioned if child unable to drink or breastfeed	15 (30.6%)	39 (43.8%)	97 (29.2%)	52 (46.8%)	74 (38.7%)	277 (35.9%)
Provider asked about normal (breast)feeding pattern	20 (40.8%)	38 (42.7%)	165 (49.7%)	21 (18.9%)	81 (42.4%)	325 (42.1%)
Provider asked about (breast)feeding pattern during this illness	12 (24.5%)	24 (27.0%)	104 (31.3%)	25 (22.5%)	53 (27.7%)	218 (28.2%)
Provider asked / caretaker mentioned diarrhea	19 (38.8%)	24 (27.0%)	49 (14.8%)	28 (25.2%)	89 (46.6%)	209 (27.1%)
Provider asked / caretaker mentioned convulsions	4 (8.2%)	10 (11.2%)	8 (2.4%)	7 (6.3%)	40 (20.9%)	69 (8.9%)
Provider asked about mother's HIV status	0 (0.0%)	1 (1.1%)	17 (5.1%)	9 (8.1%)	24 (12.6%)	51 (6.6%)
Provider counted respiration for 60 seconds	8 (16.3%)	37 (41.6%)	82 (24.7%)	20 (18.0%)	29 (15.2%)	176 (22.8%)
Provider weighed client	35 (71.4%)	66 (74.2%)	273 (82.2%)	24 (21.6%)	33 (17.3%)	431 (55.8%)
Provider plotted weight on growth chart	2 (4.1%)	3 (3.4%)	39 (11.7%)	4 (3.6%)	12 (6.3%)	60 (7.8%)
Provider took temperature	33 (67.3%)	75 (84.3%)	280 (84.3%)	81 (73.0%)	116 (60.7%)	585 (75.8%)
Provider looked into child's mouth	25 (51.0%)	13 (14.6%)	41 (12.3%)	10 (9.0%)	29 (15.2%)	118 (15.3%)
Provider checked vaccination card or vaccinated	8 (16.3%)	32 (36.0%)	124 (37.3%)	97 (87.4%)	86 (45.0%)	347 (44.9%)
Provider asked if child received vitamin A within past 6 months	0 (0.0%)	4 (4.5%)	3 (0.9%)	2 (1.8%)	3 (1.6%)	12 (1.6%)
Provider explained dosing if medication prescribed	21 (53.8%)	43 (51.2%)	165 (59.4%)	53 (50.0%)	48 (27.3%)	330 (48.3%)
Provider recommended food / liquid intake	27 (55.1%)	31 (34.8%)	190 (57.2%)	48 (43.2%)	55 (28.8%)	351 (45.5%)
Provider described ≥ 1 danger sign requiring return to facility	6 (12.2%)	9 (10.1%)	23 (6.9%)	20 (18.0%)	33 (17.3%)	91 (11.8%)
Provider discussed follow-up appt (sick child)	3(6.1%)	15 (16.9%)	115 (34.6%)	24 (21.6%)	44 (23.0%)	201 (26.0%)

Table S4. Modeling strategy for analyses

Model #	Description	Variables included
Model 0 A	Empty model no random intercept	
Model 0 B	Empty model with random intercept	
Model 1	Child and caretaker characteristics	Number of symptoms caretaker identified<br>Child gender<br>Age of child<br>Age of caretaker<br>Education level of caretaker
Model 2	Model 1 + health system foundations	Facility type<br>Facility management<br>Readiness of facility to provide child health services<br>Urban setting<br>Provider type<br>Provider gender<br>Provider years since graduation<br>Provider receives regular salary supplement
Model 3	Model 2 + Process of care	Technical quality of care observed<br>Problem with days or hours services are provided<br>Problem with wait time<br>Problem with cleanliness of facility<br>Problem with how staff treated patient<br>Problem with ability to discuss concerns with provider<br>Problem with amount of explanation provided<br>Problem with medication<br>Problem with cost of services
Model 4	Model 3 + Outer context	Countries
