# Supplementary material for: What are the determinants of variation in caretaker satisfaction with sick child consultations? A cross-sectional analysis in five low-income and middle-income countries
Source: BMJ Open. 2023 Dec 19;13(12):e071037. doi: 10.1136/bmjopen-2022-071037 (PMC10749010; doi:10.1136/bmjopen-2022-071037)
Supplement: Author's manuscript [file bmjopen-2022-071037.draft_revisions.pdf]

BMJ Open is committed to open peer review. As part of this commitment we make the peer review history of every article we publish publicly available.

When an article is published we post the peer reviewers' comments and the authors' responses online. We also post the versions of the paper that were used during peer review. These are the versions that the peer review comments apply to.

The versions of the paper that follow are the versions that were submitted during the peer review process. They are not the versions of record or the final published versions. They should not be cited or distributed as the published version of this manuscript.

BMJ Open is an open access journal and the full, final, typeset and author-corrected version of record of the manuscript is available on our site with no access controls, subscription charges or pay-per-view fees (http://bmjopen.bmj.com).

If you have any questions on BMJ Open's open peer review process please email info.bmjopen@bmj.com

BMJ Open

Determinants of variation in caretaker satisfaction with sick child consultations in five low- and middle-income countries: a multilevel analysis

Journal:	*BMJ Open*
Manuscript ID	bmjopen-2022-071037
Article Type:	Original research
Date Submitted by the Author:	16-Dec-2022
Complete List of Authors:	Turcotte-Tremblay, Anne-Marie; Université Laval; Harvard University T H Chan School of Public Health, Global Health and Population<br>Lee, Hwa-Young; Harvard University T H Chan School of Public Health<br>Kruk, Margaret; Harvard University T H Chan School of Public Health, Global Health and Population
Keywords:	Quality in health care < HEALTH SERVICES ADMINISTRATION & MANAGEMENT, Community child health < PAEDIATRICS, HEALTH SERVICES ADMINISTRATION & MANAGEMENT

SCHOLARONE™
Manuscripts

I, the Submitting Author has the right to grant and does grant on behalf of all authors of the Work (as defined in the below author licence), an exclusive licence and/or a non-exclusive licence for contributions from authors who are: i) UK Crown employees; ii) where BMJ has agreed a CC-BY licence shall apply, and/or iii) in accordance with the terms applicable for US Federal Government officers or employees acting as part of their official duties; on a worldwide, perpetual, irrevocable, royalty-free basis to BMJ Publishing Group Ltd ("BMJ") its licensees and where the relevant Journal is co-owned by BMJ to the co-owners of the Journal, to publish the Work in this journal and any other BMJ products and to exploit all rights, as set out in our licence.

The Submitting Author accepts and understands that any supply made under these terms is made by BMJ to the Submitting Author unless you are acting as an employee on behalf of your employer or a postgraduate student of an affiliated institution which is paying any applicable article publishing charge ("APC") for Open Access articles. Where the Submitting Author wishes to make the Work available on an Open Access basis (and intends to pay the relevant APC), the terms of reuse of such Open Access shall be governed by a Creative Commons licence – details of these licences and which Creative Commons licence will apply to this Work are set out in our licence referred to above.

Other than as permitted in any relevant BMJ Author's Self Archiving Policies, I confirm this Work has not been accepted for publication elsewhere, is not being considered for publication elsewhere and does not duplicate material already published. I confirm all authors consent to publication of this Work and authorise the granting of this licence.

For peer review only

Determinants of variation in caretaker satisfaction with sick child consultations in five low- and middle-income countries: a multilevel analysis

Authors: Turcotte-Tremblay, Anne-Marie[1,2], Lee, Hwa-Young[2,3]*, Kruk, Margaret E.[2]

Affiliations:

1. Faculty of Nursing, Université Laval, Laval, QC, Canada
2. Department of Global Health and Population, Harvard T.H. Chan School of Public Health, Boston, MA, United States
3. Institute of Convergence Science (ICONS), Convergence Science Academy, Yonsei University, Seoul, South Korea

*Corresponding author:

Hwa-Young Lee

401 Park Drive, 3rd Floor East, room L3-015A5
Boston MA 02115
dianna0224 @gmail.com
Telephone: 1-514-770-2386

Word count: 3 700

Keywords: user satisfaction, process of care, variance explained, multilevel modeling, low- and middle-income countries

For peer review only - http://bmjopen.bmj.com/site/about/guidelines.xhtml

ABSTRACT

Objectives: User satisfaction measures are increasingly used to evaluate the performance of healthcare services, improve accountability, and provide financial incentives to healthcare staff. However, studies have not sufficiently assessed the extent to which both health system and non-health system factors influence variation in user satisfaction in low- and middle- income countries. The objective of this study was to explore determinants of variation in caretaker satisfaction with curative care for their sick children in five low-and middle-income countries.

Methods: We used the data from the Service Provider Assessment in five countries (Afghanistan, Democratic Republic of the Congo, Haiti, Malawi, and Tanzania) to conduct a cross-sectional study between 2013–2018. The outcome variable was whether the child's caretaker was very satisfied versus not very satisfied. Predictors pertained to child and caretaker characteristics, health system foundations, and process of care (e.g., care competence, user experience). Two-level logistic regression models were used to assess the extent to which these categories of variables explained variation in satisfaction. The main analyses used pooled data; country-level analyses were also performed.

Results: Process of care accounted for the largest proportion of variance explained in user satisfaction (13.8%). In contrast, variables related to health system foundations were less important (3.8%). The final model explained only 21.8% of the total variance. Country level analyses showed differences in variance explained and in associations with predictors.

Discussion: Better process of care, especially user experience, was associated with higher caretaker satisfaction. User experience should be prioritized both as an intrinsic priority and for its instrumental benefit in terms of user satisfaction. Unmeasured factors explained the majority of variation in user satisfaction and should be explored in future studies.

Keywords: user satisfaction, process of care, variance explained, multilevel modeling, low- and middle-income countries

For peer review only - http://bmjopen.bmj.com/site/about/guidelines.xhtml

Key message

What is already known on this topic: User satisfaction is a complex and multidimensional concept with numerous determining factors. To date, however, no study has examined how much of the variation in user satisfaction with sick child consultations is explained by health system and non-health system factors at the facility- and consultation-levels in low- and middle-income countries (LMICs).

What this study adds: The process of care, including the technical quality of care and the user experience, accounts for the largest proportion of variance explained in caretaker satisfaction with sick child care across five LMICs.

How this study might affect research, practice or policy: Interventions targeting user experience may be beneficial to improve user satisfaction, thereby positively influencing utilization rates and patient retention over the course of care.

For peer review only

BACKGROUND

User satisfaction is at the heart of high-quality health systems. It reflects the ultimate judgment of the consumer and influences decisions on when and where to seek care [1]. Consequently, user satisfaction measures are increasingly used to evaluate the performance of healthcare systems, improve accountability, and provide financial incentives to healthcare staff. The results of user satisfaction surveys are also used to help managers and healthcare providers identify service factors needing improvement [2].

However, whether and to what extent user satisfaction measures are appropriate to evaluate health systems depends on the extent to which they are influenced by health system versus other factors. Studies have shown that user satisfaction is a complex and multidimensional concept with numerous determining factors. Batbaatar *et al* [2] reported that user satisfaction is influenced by technical care, interpersonal care, physical environment, access, organizational characteristics, continuity of care, and outcome of care. Focusing on child health, McCormick *et al* [3] identified the health status of the child, sociodemographic characteristics, and history of treatment as predictors of parental satisfaction in the United States (US). However, these analyses have not paid much attention to broader factors (e.g., facility characteristics, country-level characteristics) that may influence user satisfaction.

Understanding the relative contributions of different types of factors to explaining variation in user satisfaction is useful to better interpret and use results from user satisfaction surveys for quality improvement. However, few studies have examined how much variation is explained by determinants of user satisfaction in various countries. One study used data from 21 European Union countries to examine variation in user satisfaction with the health system [4]. Those authors

For peer review only - http://bmjopen.bmj.com/site/about/guidelines.xhtml

found that all factors taken into account in their model, such as patient expectations, health status, and personality, explained only 17.5% of the observed variation in user satisfaction, of which 10.4% was explained by user experience with system responsiveness [4]. They concluded that, contrary to published reports, user experience accounts for only a small fraction of variation in user satisfaction with the healthcare system. However, the proportion of variance in user satisfaction explained by health system and non-health system factors in low- and middle-income countries (LMICs) remains unclear. In addition, although it is highly likely that a number of factors affecting user satisfaction cluster at the facility level, the aforementioned study was based on single-level analyses and therefore was unable to determine how much of the variation in user satisfaction was explained at the facility level versus the consultation level.

The objectives of our study were threefold: 1) to assess the proportion of variance in caretaker satisfaction explained by factors related to child and caretaker characteristics, health system foundations, and process of care; 2) to estimate the relative proportion of variation in caretaker satisfaction attributable to the facility and consultation levels; and 3) to identify variables associated with caretaker satisfaction in five LMICs.

METHODS

Data source and sampling

The data were obtained from Service Provision Assessment (SPA) surveys. SPA surveys are conducted by the Demographic and Health Survey (DHS) Program of United States Agency for International Development (USAID), with a national statistics agency in the countries surveyed to

For peer review only - http://bmjopen.bmj.com/site/about/guidelines.xhtml

measure the capacity of health systems in LMICs. Facilities are selected from a comprehensive list of health facilities in each country, categorized by facility type, managing authority, and region; a nationally representative sample of health facilities is selected. A description of the methodology used for SPA surveys is available online [5].

We limited our study population to the most recent SPA surveys that included a question on user satisfaction with consultation for sick children in five countries: Afghanistan (2018), Democratic Republic of the Congo (DRC) (2018), Haiti (2017), Malawi (2013), and Tanzania (2015).

We used data from four separate modules: facility audits, health provider interviews, observation of protocols for sick child care, and exit interviews with caretakers. Facility audits collected data on available human resources, basic amenities, and availability of essential equipment, supplies, and medicines. A sample of health providers was selected from the facility roster to be interviewed and observed. Trained enumerators observed clinical visits to assess adherence to clinical guidelines during consultations for sick children. The caretakers (e.g., parents, grandparents) of children observed during the consultations completed an exit interview regarding their level of satisfaction with the services received, problems encountered, and demographic characteristics.

Framework

To conceptualize our analyses, we adapted the High Quality Health Systems (HQHS) framework from the Lancet Global Health Commission [1]. This framework comprises three key domains:

For peer review only - http://bmjopen.bmj.com/site/about/guidelines.xhtml

foundations, process of care, and quality impacts (Figure S1) [1]. Health system foundations include the population and their health needs, health sector governance, care delivery platforms, workforce skills, and tools such as medicines and equipment. Process of care is composed of two sub-domains: competent care and user experience. Competent care refers to systematic assessment, correct diagnosis, appropriate treatment, counseling, prevention, and detection. Positive user experience refers to respect, autonomy, clear communication, short wait times, patient affordability, and ease of use. Caretaker satisfaction, our outcome of interest, is a quality impact measure that contributes to confidence in the system and willingness to use health care.

Outcome variable

The outcome variable is caretaker satisfaction, extracted from the exit interview module. The following question was used to elicit caretaker satisfaction regarding the sick child consultation: "In general, which of the following statements best describes your opinion of the services you either received or were provided at this facility today? Very satisfied, more or less satisfied, or not satisfied." Based on the low levels of dissatisfaction, we created a binary variable of satisfaction equal to 1 if the caretaker was very satisfied and 0 otherwise.

Predictor variables

We identified child and caretaker characteristics that were likely to influence user satisfaction by reviewing previous studies [1–4]. Child characteristics included gender (male or female), age (12 months or less, 13 months or older, and age not specified), and severity of symptoms, constructed by summing the reasons the caretaker identified for bringing in the child, including cough,

For peer review only - http://bmjopen.bmj.com/site/about/guidelines.xhtml

diarrhea, fever, vomiting, problem with feeding, convulsions, excessive sleepiness, or other symptoms (range: 1~8). Caretaker characteristics included age (0–19, 20–35, ≥36 years, and age not specified) and education level (none, primary, secondary, tertiary, or higher).

To characterize the facilities, we examined the types of management authorities (public, private not-for-profit, private for-profit) and whether providers received a regular salary supplement. Facility types categorized into hospitals, health centers or clinics, health posts or dispensaries, and urban setting (versus rural) were included. To characterize the workforce, providers were separated into three categories: physicians/clinical officers/assistant medical officers; nurses/midwives; and aides/assistants. Other provider characteristics included gender and years since graduation (as a proxy for experience). To characterize the facilities' tools, we drew on data from facility audits to calculate a service readiness score for preventive and curative care regarding sick child health based on the World Health Organization (WHO) Service Availability and Readiness Assessment (SARA) guidelines [6]. The overall score was calculated by averaging all items in four domains: staff and training, equipment, diagnostics, and medicines and commodities. The original scores ranged between 0 and 1 but were multiplied by 10 to facilitate interpretation (0–10). Table S1 presents the full list of indicators used (see supplementary file).

We assessed the technical quality of sick child consultations by investigating the degree to which the provider adhered to clinical guidelines during the consultation observation [7,8]. The technical quality score was calculated as the percentage of clinically recommended tasks performed in four domains: patient history-taking, physical exam, drug administration and immunization, and client education and counseling. We also multiplied the score by 10 to facilitate interpretation. The items included for technical quality indicators for sick child care are presented in the supplementary file (Table S2 and Table S3).

Caretakers' perceptions of their experience during the visit were examined through exit interviews by asking whether they had encountered problems that day related to: 1) days or hours of service at this facility (i.e., when it opened and closed); 2) wait time to see a provider; 3) facility cleanliness; 4) how staff treated them; 5) ability to discuss problems or concerns about the health issue; 6) amount of explanation received about the problem or treatment; 7) medication; and 8) cost of services or treatments. For each of these, responses were scored as the number of problems encountered. We included the country variables as a proxy for country-level outer context.

For peer review only - http://bmjopen.bmj.com/site/about/guidelines.xhtml

Statistical analysis

We calculated descriptive statistics for all variables of interest, presenting the proportions for categorical variables and mean and standard deviation (SD) for continuous covariates. These descriptive analyses incorporated survey sampling weights for patients and facilities.

For the main analyses, we performed a two-level logistic regression analysis based on pooled data from the five countries, where level 1 was the consultation and level 2 was the facility. This provided variance estimates while accounting for clustering effect at the facility level. The following general structure was used:

$$logit\ (\Pr\ (Y_{ji} = 1|X) = \beta_0 + \beta_1 \cdot X_{ij} + u_{0j}$$

where Y (caretaker satisfaction) and X (representing a vector of independent variables) were each assumed to follow a two-level data structure, with both fixed-effects ($\beta_0 + \beta_1 \cdot X_{ij}$) and random-effects parameters (u_{0j}). The random-effects parameter (u_{0j}) was assumed to follow a normal distribution, with mean of 0 and variances of u_{0j} ~ N (0, $\sigma^2_{u_0}$).

Four models were estimated based on the general modeling structure outlined above, by incrementally adding blocks of covariates following our conceptual framework (see Table S4 in the supplementary file for details on the modeling strategy). M0 was a null/unadjusted model with an intercept term in the fixed part of the model. M1 added child and caretaker characteristics. M2 built on the previous model to include variables related to health system foundations. M3 added variables related to the process of care. For the analyses with the pooled data, M4 included the country as a variable.

We applied Snijders and Bosker's method to measure the explained proportion of variance in caretaker satisfaction for the logistic multilevel model [9]. We assumed that the dichotomous

For peer review only - http://bmjopen.bmj.com/site/about/guidelines.xhtml

outcome Y could be conceived as generated through a threshold model with underlying variable $\check{Y}$, denoted as:

$$\check{Y}_{ij} = \gamma^0 + \sum_{h=1}^{\gamma} \gamma^h X_{hji} + U_{0j} + R_{ij}$$

Hence the total variance of Y_{ij} was equal to:

$$\text{Var}\ (Y_{ij}) = \sigma_F^2 + \tau_0^2 + \sigma_R^2$$

where σ_F^2 is the explained variance, which is the observed variance of the linear predictor $\hat{Y}_{ij} = \gamma^0 + \sum_{h=1}^{\gamma} \gamma^h X_{hji}$, and τ_0^2 and σ_R^2 are unexplained variances at levels 2 and 1, which were var (U_{0j}) and var (R_{ij}), respectively. The proportion of total variance explained by the fixed effects in the model, corresponding to an R^2 equivalent for multilevel logistic regression, is defined by:

$$R_{dicho}^2 = \frac{\sigma_F^2}{\sigma_F^2 + \tau_0^2 + \sigma_R^2}$$

Since logistic regression models do not have a level 1 residual term, unexplained variance at the consultation level was estimated as $\pi^2/3 \approx 3.29$ based on the method summarized by Goldstein *et al* (8). When considering only the unexplained part of the total variance, the proportion attributed to the health facilities level (called residual intraclass correlation) was evaluated as:

$$\rho_I = \frac{\tau_0^2}{\tau_0^2 + \sigma_R^2}$$

We also performed country-stratified analyses to examine whether the results were country-specific. All analyses were conducted in Stata 16.0 (StataCorp, College Station, Texas).

Patient and public involvement statement

For peer review only - http://bmjopen.bmj.com/site/about/guidelines.xhtml

Patients and/or the public were not involved in the design, or conduct, or reporting, or dissemination of this research.

Ethical consideration

SPA data are publicly accessible from the DHS website. Participants provided consent for the original data collection. This secondary analysis of deidentified data was deemed exempt from human subjects review.

RESULTS

Descriptive statistics

Table 1 presents the demographic characteristics and healthcare situations in the five countries included. All of these countries are characterized by high rates of neonatal mortality. DRC had the highest mortality rate for children under the age of five per 1,000 live births (58) and the lowest health spending per capita (51 PPP$). In contrast, Malawi had the lowest mortality rate for children under the age of five (39) and the second lowest value for health spending per capita (82 PPP$).

For peer review only

Table 1. Context of study countries

	Afghanistan	DRC	Haiti	Malawi	Tanzania
Population (in millions)*	38.0	86.8	11.3	18.6	58.0
Urban population (% of total population)†	26	46	57	17	35
Gross domestic product per person (USD)*	507	581	1,273	412	1,122
Health expenditure per capita (PPP$)*	286	51	143	82	99
Primary education completion rate, total (% of relevant group)‡	84	70	49	80	69
Literacy rate of adults (%) ‡§	43	77	62	62	78
Birth attended by skilled health staff (%)‡	59	80	42	90	64
Maternal mortality (per 100,000 live births)**	638	473	480	349	524
Neonatal mortality (per 1,000 live births)*	36	27	25	20	20
Mortality rate under age five (per 1,000 live births)†	58	81	61	39	49
Hospital beds (per 1,000 people)‡	0.4	0.8	0.7	1.3	0.7

* Data from 2019. † Data from 2020. ‡ Data from the most recent year available.

§ Percentage of people aged 15 and above able to read & write simple statements about everyday life.

** Data from 2017.

We performed a complete case analysis, which yielded a total of 13,149 sick child visits across 3,421 facilities for the analytic sample in the five countries (Table 2). For variables with the most missing data (e.g., child age, caretaker age), we added the value "not specified" to be able to include the cases in the analysis (Table 2). Less than 5% of the sample had missing data, in total.

Table 2 presents summary statistics for the analytic sample. Overall, 77% of caretakers were very satisfied. The percentage of very satisfied respondents was highest in Haiti (89%) and lowest in Afghanistan (62%). Approximately 68% of caretakers had primary education level or less. Of the providers, 34% were female, and 58% were qualified as doctors or advance practice clinicians. The majority of facilities were health centers (52%) and public (63%). The mean scores for technical quality of care observed were quite low (3.1). Because data in Afghanistan only cover

For peer review only - http://bmjopen.bmj.com/site/about/guidelines.xhtml

urban areas, the percentage of highly qualified providers, hospitals, and private for-profit facilities was notably higher than in the other countries (Table 2).

For peer review only

For peer review only - http://bmjopen.bmj.com/site/about/guidelines.xhtml

Table 2. Descriptive statistics per country

Variables	Afghanistan N (%)	DRC N (%)	Haiti N (%)	Malawi N (%)	Tanzania N (%)	Total N (%)
Very satisfied/total analytic sample, N (very satisfied, %)	345/556 (62)	1859/2592 (71)	1850/2093 (89)	2581/3177 (82)	3538/4731 (75)	10171/13149 (77)
Child and caretaker characteristics						
Number of symptoms caregiver identified (0-8), Mean (SD)	3.2 (1.3)	3.4 (1.4)	2.9 (1.4)	2.5 (1.2)	2.7 (1.3)	2.8 (1.3)
Child is female	236 (43%)	1238 (47%)	974 (47%)	1566 (50%)	2333 (49%)	6346 (48%)
Age of child (months)						
12 or less	263 (47%)	959 (37%)	1026 (49%)	1300 (41%)	1974 (42%)	5520 (42%)
13 or above	246 (44%)	1618 (62%)	1045 (50%)	1833 (58%)	2768 (58%)	7508 (57%)
not specified	47 (8%)	33 (1%)	15 (1%)	21 (1%)	8 (0%)	123 (1%)
Age of caretaker (yrs)						
20-35	366 (66%)	1799 (69%)	1489 (71%)	2471 (78%)	3505 (74%)	9628 (73%)
0-19	19 (3%)	222 (8%)	166 (8%)	324 (10%)	439 (9%)	1167 (9%)
36+	110 (20%)	424 (16%)	407 (20%)	249 (8%)	719 (15%)	1906 (14%)
not specified	62 (11%)	167 (6%)	24 (1%)	111 (3%)	87 (2%)	449 (3%)
Education of caretaker						
None	308 (55%)	569 (22%)	231 (11%)	385 (12%)	901 (19%)	2392 (18%)
Primary	53 (9%)	828 (32%)	583 (28%)	2021 (64%)	3072 (65%)	6556 (50%)
Secondary	121 (22%)	1065 (41%)	1105 (53%)	683 (22%)	693 (15%)	3665 (28%)
Tertiary or higher	74 (13%)	149 (6%)	167 (8%)	66 (2%)	83 (2%)	538 (4%)
Health system foundations						
Provider is female	14 (3%)	576 (14%)	991 (60%)	568 (29%)	2288 (45%)	4435 (34%)
Provider type						
Doctor, advanced practice clinician, paramedical	460 (100%)	1225 (31%)	1196 (72%)	1631 (83%)	3112 (61%)	7622 (58%)
Nurse, midwife	0 (0%)	2689 (67%)	463 (28%)	324 (16%)	1938 (38%)	5413 (41%)
Othes - Pharm, lab, dental, non-clinical	0 (0%)	90 (2%)	4 (0%)	11 (1%)	12 (0%)	115 (1%)
Provider receives regular salary supplements	282 (61%)	579 (14%)	168 (10%)	746 (38%)	1461 (29%)	3234 (25%)
Service readiness score for sick child care (0-10), Mean (SD)	6.0 (1.3)	6.5 (1.8)	5.7 (1.6)	6.0 (1.6)	5.2 (1.6)	5.8 (1.7)
Facility type						
Hospitals	353 (61%)	520 (22%)	558 (23%)	591 (16%)	288 (7%)	2308 (18%)
Health centers	227 (39%)	1861 (78%)	1283 (53%)	2804 (78%)	627 (15%)	6800 (52%)
Health posts and dispensaries	0 (0%)	0 (0%)	563 (23%)	195 (5%)	3285 (78%)	4042 (31%)
Facility management						
Public	157 (27%)	1506 (63%)	800 (33%)	2334 (65%)	3486 (83%)	8281 (63%)
Private not-for-profit	71 (12%)	576 (24%)	906 (38%)	748 (21%)	383 (9%)	2682 (20%)
Private for-profit	352 (61%)	299 (13%)	697 (29%)	509 (14%)	332 (8%)	2187 (17%)
Urban facility	575 (99%)	446 (19%)	1128 (47%)	749 (21%)	1070 (25%)	3966 (30%)
Process of care						
Technical quality of care observed (0-10), mean (SD)	2.4 (1.1)	3.4 (1.2)	3.1 (1.2)	3.0 (1.3)	3.1 (1.5)	3.1 (1.4)

For peer review only - http://bmjopen.bmj.com/site/about/guidelines.xhtml

Problem with days or hours services are provided	73 (13%)	326 (12%)	128 (6%)	601 (19%)	612 (13%)	1738 (13%)
Problem with wait time	137 (25%)	530 (20%)	474 (23%)	998 (32%)	1329 (28%)	3466 (26%)
Problem with cleanliness of facility	92 (16%)	481 (18%)	100 (5%)	250 (8%)	769 (16%)	1690 (13%)
Problem with how staff treated patient	100 (18%)	269 (10%)	48 (2%)	229 (7%)	317 (7%)	961 (7%)
Problem with ability to discuss concerns with provider	77 (14%)	323 (12%)	180 (9%)	354 (11%)	397 (8%)	1328 (10%)
Problem with amount of explanation provided	75 (13%)	372 (14%)	163 (8%)	356 (11%)	401 (8%)	1365 (10%)
Problem with medication availability	156 (28%)	581 (22%)	205 (10%)	635 (20%)	1540 (32%)	3115 (24%)
Problem with cost of services	193 (35%)	575 (22%)	105 (5%)	174 (6%)	462 (10%)	1508 (11%)

For peer review only

For peer review only - http://bmjopen.bmj.com/site/about/guidelines.xhtml

Total variance explained and residual intraclass correlation

Figure 1 shows the percentage of variance explained in each model. Variance explained in the fully adjusted model with the combined data from five countries was 21.8%. However, it varied significantly across countries, ranging from 9.0% in DRC to 51.7% in Afghanistan. Factors related to patient and caretaker characteristics only explained 0.9% of the total variance (0.8% in Tanzania ~ 4.3% in Afghanistan). The explained variance increased by 3.8% when variables related to health system foundations, such as facility and provider characteristics, were included (0.9% in Malawi ~ 10.8% Afghanistan). The largest percentage of the total variance was explained by process of care in both the pooled and country-stratified analyses. When process of care variables were added, the explained variance increased by 13.8% in the pooled analyses. Variance explained by process of care was notably higher in Afghanistan and Tanzania (36.6% and 24.2%, respectively) than in the other countries (5.8% in DRC ~ 10.4% in Malawi).

[INSERT Figure 1. Variance explained with addition of variables to the model]

Figure 2 indicates the percentage of unexplained variance attributed to each level in the null and final models. The percentage of variance attributable to the facility level in the unadjusted model was highest in Afghanistan (44.0%) and lowest in Haiti (22.7%). Factors considered in our study explained the most variance at the facility in Afghanistan (21.6%) while they explained only 1.6% of variance at facility level in DRC.

[INSERT Figure 2. Residual intraclass correlation (% of unexplained variance)]

For peer review only - http://bmjopen.bmj.com/site/about/guidelines.xhtml

Determinants of caretaker satisfaction

Table 3 shows the results of the final model's fixed part based on the pooled data from the five countries. More severe symptoms of the child (OR: 0.94, 95% CI: 0.91 to 0.98) and higher education of caretakers (OR: 0.78 and 0.53, 95% CI: 0.66 to 0.93 and 0.40 to 0.69 for secondary and tertiary level or higher, respectively) were associated with lower odds of being very satisfied with the service received. Caretakers who sought care in hospital were less likely to be very satisfied with service received than those at lower-level facilities. In particular, caretakers who visited health posts and dispensaries for their sick child were 1.82 times more likely to be very satisfied with the service than those who visited hospitals (OR: 1.33; 95% CI: 1.11 to 1.59 and OR: 1.82; 95% CI: 1.40 to 2.38, respectively). Caretakers whose child received care in facilities with higher service readiness scores were more likely to be very satisfied.

Problems with process of care were strong predictors of user satisfaction. Higher technical quality scores for sick child care increased the likelihood of being very satisfied (OR: 1.08, 95% CI: 1.04 to 1.13 per 10% increase). Negative user experiences significantly decreased the odds of caretaker satisfaction. In particular, caretakers who perceived they were not given adequate explanation and perceived a problem with medication availability were 0.56 and 0.31 times less likely to be very satisfied with the service for their sick child, respectively (95% CI: 0.46 to 0.67 and 0.27 to 0.35, respectively). Problems with the cost of services significantly reduced the odds of caretaker satisfaction (OR: 0.57, 95% CI: 0.48 to 0.66). None of the providers' characteristics were associated with caretaker satisfaction. All country dummy variables, which served as proxies for national context, were significant. Compared to caretakers in Malawi, those in Afghanistan,

For peer review only - http://bmjopen.bmj.com/site/about/guidelines.xhtml

DRC, and Tanzania were less likely to be very satisfied with services, while those in Haiti were 1.76 times more likely to be very satisfied.

For peer review only

For peer review only - http://bmjopen.bmj.com/site/about/guidelines.xhtml

Table 3. Determinants of caretaker satisfaction

Variables	Odd ratio		95% CI	
Child and caretaker characteristics				
Number of symptoms caregiver identified	0.94	**	0.91,	0.98
Child is female	0.95	ns	0.86,	1.05
Age of child (month) (ref: 12 or less)				
13 or older	1.03	ns	0.93,	1.15
not specified	0.69	ns	0.41,	1.15
Age of caretaker (yrs) (ref: 20-35)				
0-19	1.17	ns	0.96,	1.42
36+	1.15	ns	0.99,	1.33
not specified	0.73	*	0.54,	0.99
Education level of caretaker (ref: none)				
Primary	0.89	ns	0.76,	1.04
Secondary	0.78	**	0.66,	0.93
Tertiary or higher	0.53	***	0.40,	0.69
Health system foundations: facility characteristics				
Types (ref: hospitals)				
Health centers	1.33	**	1.11,	1.59
Health posts and dispensaries	1.82	***	1.40,	2.38
Facility management (ref: public)				
Private not-for-profit	1.06	ns	0.90,	1.27
Private for-profit	1.09	ns	0.87,	1.36
Readiness score for sick child care (per 10% increase)	1.08	***	1.03,	1.13
Urban facility (ref: rural)	0.92		0.78,	1.09
Health system foundations: provider characteristics				
Types (ref: Doctor, advanced practice clinician, paramedical)				
Nurse, midwife	0.96	ns	0.80,	1.14
Other - Pharm, lab, dental, non-clinical	1.27	ns	0.65,	2.52
Provider is female (ref: male)	1.02	ns	0.88,	1.17
Years since graduation or completion of qualification	1.00	ns	0.99,	1.00
Receiving regular salary supplements (ref: no)	1.05	ns	0.90,	1.22
Process of care				
Technical quality of care (per 10% increase)	1.08	***	1.04,	1.13
Problem with~				
Days or hours of services provided (ref: no)	0.90	ns	0.77,	1.06
Waiting time (ref: no)	0.61	***	0.54,	0.68
Cleanliness of facility (ref: no)	0.83	*	0.71,	0.98
How staff treated patient (ref: no)	0.60	***	0.50,	0.73
Ability to discuss concerns with provider (ref: no)	0.95	ns	0.78,	1.17
Amount of explanation provided (ref: no)	0.56	***	0.46,	0.67
Medication availability (ref: no)	0.31	***	0.27,	0.35
Cost of services (ref: no)				
Yes	0.57	***	0.48,	0.66
Not specified	0.93		0.61,	1.43
Outer context				
Country (ref: Malawi)				
Afghanistan	0.37	***	0.24,	0.57
DRC	0.58	***	0.46,	0.74
Haiti	1.76	***	1.35,	2.29
Tanzania	0.58	***	0.47,	0.71

* $p \leq 0.05$; ** $p \leq 0.01$; *** $p \leq 0.001$

For peer review only - http://bmjopen.bmj.com/site/about/guidelines.xhtml

The results of the fixed country effects are presented in a supplementary file (Table S5). Severity of child symptoms and private for-profit facilities were significantly associated with satisfaction only in DRC. On the other hand, in DRC some aspects of the patient experience, such as wait time and how staff treated patients, appeared to be less important predictors of caretaker satisfaction.

DISCUSSION

To our knowledge, this is the first multi-country study to examine factors affecting variation in caretaker satisfaction with sick child consultations in multiple LMICs. Several findings deserve attention. First and foremost, in both the pooled and country-stratified analyses, user-reported problems with process of care accounted for the largest proportion of variance explained in caretaker satisfaction with sick child care. The proportion of variance explained by factors pertaining to health system foundations was considerably less significant. Patient and caretaker characteristics explained the least proportion of variance. This finding is consistent with two previous studies. In Peru, Leslie *et al* [10] found that user experience accounted for a larger proportion of variance in all user satisfaction measures, including three and five categories of net promoter score (NPS) and satisfaction level, than the proportion explained by individual, facility, and contextual factors combined. Similarly, Bleich *et al*'s study [4] based on 21 European Union countries reported that all variables considered in the analyses (e.g., user experience, user expectations) accounted for 17.5% of the observed variation in user satisfaction, of which 10% was explained by user experience.

For peer review only - http://bmjopen.bmj.com/site/about/guidelines.xhtml

Second, technical quality of care, a sub-dimension of process of care, was a significant predictor of caretaker satisfaction with the service. Odds of caretakers' being very satisfied with the service increased 1.08 times with every 10% increase in technical quality score—a substantial effect size if quality were 30% or 40% higher, which is conceivable given low baseline performance. However, this association was not significant in three of the five countries. The reason for this may be that, in some contexts, patients find it difficult to assess technical quality of care due to lack of medical expertise [11]. In contrast, it may be easier for patients to perceive tangible aspects, such as outcome quality (e.g., how much the symptoms have been improved) [12,13], and assess their user experience (e.g., wait times, affordability of care) [14–16]. This should be taken into account when user satisfaction is used to assess the performance of facilities.

Third, we found substantial between-country heterogeneity. The proportion of the total variance explained by the variables was notably higher in Afghanistan (51.7 %, as opposed to 9.0%~28.6% in the other four countries). Also, the proportion of variance explained by process of care was highest in Afghanistan (increase in R squared: 36.6% in Afghanistan vs. 5.8%~24.2%). Compared to other countries, the provider's courtesy and communication (e.g., explanations, discussions) appeared to be particularly important aspects of caretaker satisfaction in Afghanistan. In DRC, total variance explained by the variables was only 9%, suggesting there were still more variables influencing caretaker satisfaction, which we were unable to take into account (e.g., insecurity, unpaid government employees). According to Bleich *et al* [4], external factors such as media coverage could influence how satisfied people are with their healthcare system. Future qualitative and mixed methods studies could be useful to explore and uncover new context-specific factors that affect variance in user satisfaction.

Factors predicting patient satisfaction also differed between countries. Experiences of problems with medication availability or cost were the only indicators related to user experience that had a marked negative association with caretaker satisfaction across all five countries. On the other hand, inability to discuss concerns with providers and problems with how staff treated

For peer review only - http://bmjopen.bmj.com/site/about/guidelines.xhtml

patients showed significant negative association with caretaker satisfaction in only a few countries, whereas it was identified as a strong predictor of user satisfaction in earlier studies conducted on different types of maternal care in other countries. Specifically, Dzomeku found that providers' unfriendliness and ineffective communication negatively affected maternal satisfaction in Ghana [17]. Similar results were found in Lebanon and Gambia, where it was reported that women were more satisfied with services when they had the providers' attention and adequate communication with them [18,19]. As such, factors linked to patient satisfaction may depend on context, which is related to various expectations, public sentiment, values, or norms [2,20]. These findings can inform strategic priorities in each country aimed at enhancing patient satisfaction with sick child consultations and possibly patient retention. The influence of cultural differences on patient satisfaction needs to be explored in future research.

This study has several limitations. First, the satisfaction rates reported by caretakers were generally high, possibly due to desirability bias in participants' responses during interviews, low patient expectations, and behaviors during the audits. Second, some potential predictors of caretaker satisfaction, such as patients' and providers' personalities and past experiences with the healthcare system, were not available from the SPA surveys and thus were not included in our analysis. Finally, because this is a cross-sectional analysis, the associations identified cannot be interpreted causally. Despite this limitation, our study has the advantage of combining multiple sources of data (facility audits, health provider interviews, exit interviews with caretakers). It is also the first multi-country analysis to examine the determinants of variation in caretaker satisfaction for sick child care in LMICs.

CONCLUSION

High-quality healthcare systems should be able to satisfy users, including caretakers of sick children. People who are satisfied may have more confidence in their healthcare system and be

For peer review only - http://bmjopen.bmj.com/site/about/guidelines.xhtml

more likely to seek care. In this regard, it is important to understand which factors shape variation in user satisfaction. Interventions targeting patient experience may be effective to improve patient satisfaction, thereby positively influencing utilization rates and patient retention over the course of care. However, the aspects of patient experience that should be prioritized may depend on the country. Additionally, there are still unidentified factors affecting variation in patient satisfaction. Future research should explore new determinants of patient satisfaction throughout children's care pathways.

For peer review only

For peer review only - http://bmjopen.bmj.com/site/about/guidelines.xhtml

ACKNOWLEDGEMENTS

The authors express their gratitude to all actors involved in collecting and reporting Service Provider Assessment data in the participating countries.

FUNDING

We acknowledge funding from the Bill & Melinda Gates Foundation (grants INV-005254). The first author received a fellowship from the Fonds de recherche du Québec – Santé. These *funding sources had no role in the design of this study* the analyses or interpretation of the data.

CONTRIBUTORSHIP STATEMENT

AMTT developed the idea, conducted the analyses and wrote the first draft of the manuscript. HYL validated the results and reviewed the manuscript. MEK helped develop the original idea, provided the database, oversaw the analyses and reviewed the manuscript.

ETHICAL APPROVAL

The original survey implementers obtained ethical approvals for data collection; the Harvard University Human Research Protection Program approved this secondary analysis as exempt from human subjects review.

COMPETING INTERESTS

The authors declare no competing interests.

DATA AVAILABILITY STATEMENT

All of the data used are publicly available through the Demographic and Health Survey (DHS) Program of United States Agency for International Development (USAID). The data can be accessed via this website: https://dhsprogram.com/Methodology/survey-search.cfm?pgtype=main&SrvyTp=year

For peer review only

REFERENCES

1 Kruk ME, Gage AD, Arsenault C, *et al.* High-quality health systems in the Sustainable Development Goals era: time for a revolution. *The Lancet Global Health* 2018;**6**:e1196–252. doi:10.1016/S2214-109X(18)30386-3

2 Batbaatar E, Dorjdagva J, Luvsannyam A, *et al.* Determinants of patient satisfaction: a systematic review. *Perspect Public Health* 2017;**137**:89–101. doi:10.1177/1757913916634136

3 McCormick MC, Escobar GJ, Zheng Z, *et al.* Factors influencing parental satisfaction with neonatal intensive care among the families of moderately premature infants. *Pediatrics* 2008;**121**:1111–8. doi:10.1542/peds.2007-1700

4 Bleich SN, Ozaltin E, Murray CKL. How does satisfaction with the health-care system relate to patient experience? *Bull World Health Organ* 2009;**87**:271–8. doi:10.2471/blt.07.050401

5 DHS Program. SPA Overview. https://dhsprogram.com/Methodology/Survey-Types/SPA.cfm

6 World Health Organization. Service availability and readiness assessment (SARA): an annual monitoring system for service delivery: reference manual. Geneva: : World Health Organization 2013.

7 World Health Organization. Partnership for maternal and newborn health. Opportunities for Africas newborns: practical data policy and programmatic support for newborn care in Africa. Geneva: : World Health Organization 2006.

8 Gera T, Shah D, Garner P, *et al.* Integrated management of childhood illness (IMCI) strategy for children under five. *Cochrane Database Syst Rev* 2016;**6**. doi:10.1002/14651858.CD010123.pub2

9 Snijders T, Bosker R. *Multilevel analysis. An introduction to basic and advanced multilevel modeling*. 2nd edition. London: : Sage Publications 2012.

For peer review only

For peer review only - http://bmjopen.bmj.com/site/about/guidelines.xhtml

10 Leslie HH, Lee H-Y, Blouin B, *et al.* Evaluating patient-reported outcome measures in Peru: a cross-sectional study of satisfaction and net promoter score using the 2016 EnSuSalud survey. *BMJ Qual Saf* 2022;**31**:599–608. doi:10.1136/bmjqs-2021-014095

11 Hanefeld J, Powell-Jackson T, Balabanova D. Understanding and measuring quality of care: dealing with complexity. *Bull World Health Organ* 2017;**95**:368–74. doi:10.2471/BLT.16.179309

12 Schaal T, Schoenfelder T, Klewer J, *et al.* Effects of perceptions of care, medical advice, and hospital quality on patient satisfaction after primary total knee replacement: a cross-sectional study. *PLoS One* 2017;**12**:e0178591. doi:10.1371/journal.pone.0178591

13 Naik J RK, Byram A. Healthcare service quality effect on patient satisfaction and behavioural intentions in corporate hospitals in India. *International Journal of Pharmaceutical and Healthcare Marketing* 2016;**10**:48–74. doi:10.1108/IJPHM-07-2014-0043

14 Srivastava A, Avan BI, Rajbangshi P, *et al.* Determinants of women's satisfaction with maternal health care: a review of literature from developing countries. *BMC Pregnancy Childbirth* 2015;**15**:97. doi:10.1186/s12884-015-0525-0

15 Bazant ES, Koenig MA. Women's satisfaction with delivery care in Nairobi's informal settlements. *Int J Qual Health Care* 2009;**21**:79–86. doi:10.1093/intqhc/mzn058

16 George A. Quality of reproductive care in private hospitals in Andhra Pradesh: women's perception. *Economic and Political Weekly* 2002;**37**:1686–92.

17 Dzomeku V. Maternal satisfaction with care during labour: a case-study of the Mampong-Ashanti District Hospital maternity unit in Ghana. *International Journal of Nursing and Midwifery* 2011;**3**:30–4.

For peer review only

For peer review only - http://bmjopen.bmj.com/site/about/guidelines.xhtml

18 Kabakian-Khasholian T, Campbell O, Shediac-Rizkallah M, *et al.* Women's experiences of maternity care: satisfaction or passivity? *Soc Sci Med* 2000;**51**:103–13. doi:10.1016/s0277-9536(99)00443-8

19 Jallow IK, Chou Y-J, Liu T-L, *et al.* Women's perception of antenatal care services in public and private clinics in the Gambia. *Int J Qual Health Care* 2012;**24**:595–600. doi:10.1093/intqhc/mzs033

20 Sanchez-Piedra CA, Jaruseviciene L, Prado-Galbarro FJ, *et al.* Factors associated with professional satisfaction in primary care: esults from EUprimecare project. *Eur J Gen Pract* 2017;**23**:114–20. doi:10.1080/13814788.2017.1305350

For peer review only

For peer review only - http://bmjopen.bmj.com/site/about/guidelines.xhtml

Figure 1. Percentage of variance explained with addition of variables to the model

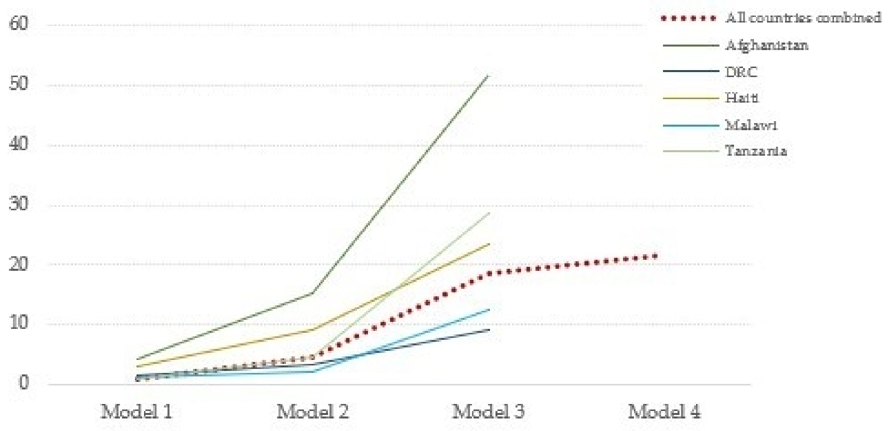

	All countries combined	Afghanistan	DRC	Haiti	Malawi	Tanzania
Null model	0.0	0.0	0.0	0.0	0.0	0.0
Model 1	0.9	4.3	1.5	3.1	1.3	0.8
Model 2	4.7	15.1	3.2	9.2	2.2	4.4
Model 3	18.5	51.7	9.0	23.6	12.6	28.6
Model 4	21.8	NA	NA	NA	NA	NA

145x132mm (96 x 96 DPI)

Figure 2. Residual intraclass correlation (% of unexplained variance)

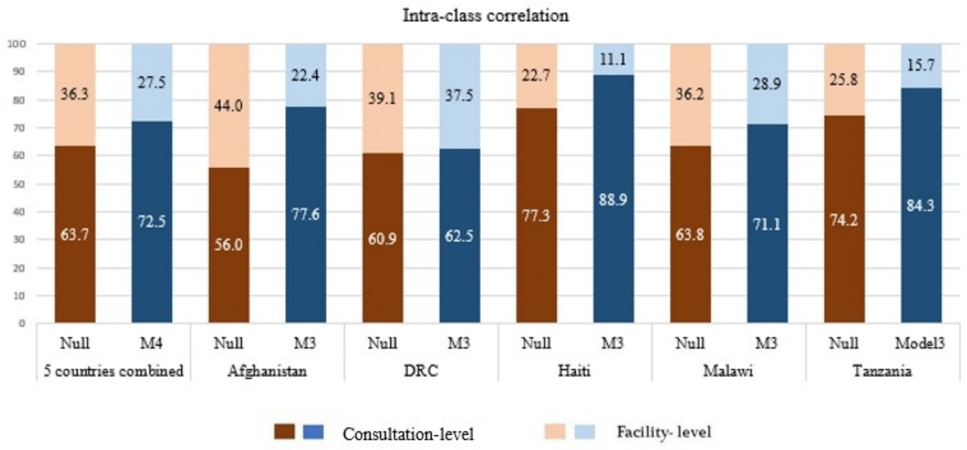

207x108mm (96 x 96 DPI)

Supplementary Files

Figure S1. Conceptual framework of health system quality

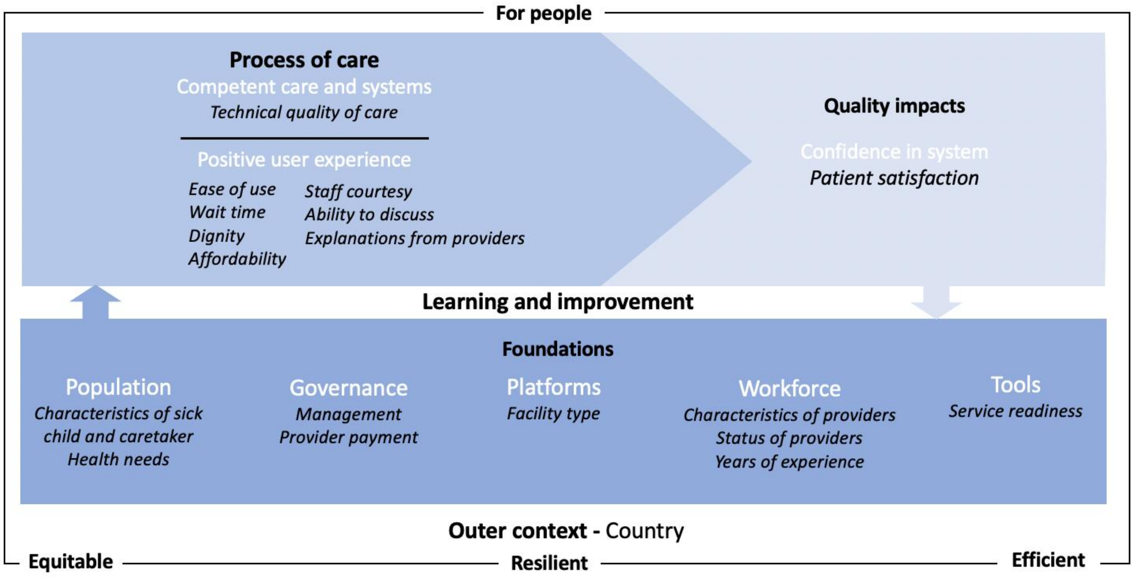

Adapted with permission from Kruk, Gage, Arsenault, et al., 2018

Table S1. Items included in the service readiness indicator for preventive and curative child health services

	Afghanistan (N = 556)	DRC (N = 2592)	Haiti (N = 2093)	Malawi (N = 3177)	Tanzania (N = 4731)	Total (N = 13149)
Staff and training						
Guidelines for IMCI	90 (16.2%)	1408 (54.3%)	483 (23.1%)	1364 (42.9%)	3006 (63.5%)	6351 (48.3%)
Guidelines for growth monitoring	86 (27.5%)	1057 (58.9%)	599 (44.9%)	1089 (38.5%)	1444 (32.8%)	4275 (40.1%)
Staff trained in IMCI	113 (20.3%)	1575 (60.8%)	716 (34.2%)	1041 (32.8%)	1430 (30.2%)	4875 (37.1%)
Equipment						
Child and infant scale	505 (90.8%)	2347 (90.5%)	1775 (84.8%)	2714 (85.4%)	3085 (65.2%)	10426 (79.3%)
Length/height measuring equipment	154 (49.2%)	1274 (70.9%)	1162 (87.2%)	2546 (90.0%)	1865 (42.4%)	7001 (65.6%)
Thermometer	420 (75.5%)	2482 (95.8%)	1975 (94.4%)	2741 (86.3%)	3991 (84.4%)	11609 (88.3%)
Stethoscope	551 (99.1%)	2426 (93.6%)	2051 (98.0%)	2932 (92.3%)	4374 (92.5%)	12334 (93.8%)
Growth chart	123 (39.3%)	1592 (88.6%)	691 (51.8%)	1785 (63.1%)	3191 (72.5%)	7382 (69.2%)
Diagnostics						
Haemoglobin / anemia testing	389 (70.0%)	2039 (83.3%)	332 (19.9%)	806 (28.6%)	2348 (65.1%)	5914 (53.3%)
Test for parasite in stool (microscopy)	446 (80.2%)	1895 (77.4%)	1456 (87.1%)	773 (27.5%)	2236 (62.0%)	6806 (61.3%)
Malaria testing	464 (83.5%)	2316 (94.6%)	1600 (95.7%)	2522 (89.6%)	3286 (91.1%)	10188 (91.8%)
Medicines and commodities						
Oral rehydration	496 (89.2%)	2092 (81.5%)	1427 (68.2%)	2899 (91.5%)	4021 (85.2%)	10935 (83.4%)
Amoxicillin syrup or tablets	528 (95.0%)	2167 (84.4%)	1973 (94.3%)	2949 (93.1%)	4197 (88.9%)	11814 (90.2%)
Co-trimoxazole syrup or suspension	442 (79.5%)	1339 (52.2%)	1560 (74.5%)	2379 (75.1%)	3683 (78.0%)	9403 (71.8%)
Paracetamol syrup or suspension	493 (88.7%)	1188 (46.3%)	1720 (82.2%)	2304 (72.7%)	3134 (66.4%)	8839 (67.5%)
Vitamin A capsule	470 (84.5%)	1170 (45.6%)	1258 (60.1%)	1477 (46.6%)	3119 (66.1%)	7494 (57.2%)
Albendazole or mebendazole	488 (87.8%)	2174 (84.7%)	1861 (88.9%)	3062 (96.7%)	4227 (89.6%)	11812 (90.1%)
Zinc tablet or syrup	409 (73.6%)	1729 (67.4%)	919 (43.9%)	2722 (85.9%)	2578 (54.6%)	8357 (63.8%)

For peer review only

Table S2. Items included in the technical quality indicator for children older than 2 months

	Afghanistan (N = 516)	DRC (N = 2452)	Haiti (N = 1799)	Malawi (N = 3029)	Tanzania (N = 4516)	Total (N = 12312)
Provider asked / caretaker mentioned if child unable to drink or breastfeed	70 (13.8%)	526 (21.0%)	319 (18.1%)	848 (27.7%)	1348 (29.7%)	3111 (25.1%)
Provider asked / caretaker mentioned cough or difficult breathing	337 (66.5%)	1576 (63.0%)	1223 (69.4%)	2206 (72.0%)	3498 (77.0%)	8840 (71.4%)
Provider asked / caretaker mentioned diarrhea	203 (40.0%)	1105 (44.1%)	648 (36.8%)	1273 (41.5%)	2813 (62.0%)	6042 (48.8%)
Provider asked / caretaker mentioned fever	384 (75.7%)	2251 (89.9%)	1274 (72.3%)	2465 (80.4%)	4216 (92.9%)	10590 (85.6%)
Provider asked / caretaker mentioned vomiting	180 (35.5%)	1084 (43.3%)	486 (27.6%)	1134 (37.0%)	2352 (51.8%)	5236 (42.3%)
Provider asked / caretaker mentioned convulsions	32 (6.3%)	259 (10.3%)	69 (3.9%)	285 (9.3%)	1041 (22.9%)	1686 (13.6%)
Provider asked about mother's HIV status	0 (0.0%)	36 (1.4%)	36 (2.0%)	216 (7.0%)	377 (8.3%)	665 (5.4%)
Provider asked / caretaker mentioned ear pain	54 (10.7%)	130 (5.2%)	102 (5.8%)	161 (5.3%)	767 (16.9%)	1214 (9.8%)
Provider counted respiration for 60 seconds	79 (15.6%)	691 (27.6%)	366 (20.8%)	501 (16.3%)	644 (14.2%)	2281 (18.4%)
Provider weighed client	240 (47.3%)	1903 (76.0%)	1446 (82.1%)	638 (20.8%)	673 (14.8%)	4900 (39.6%)
Provider plotted weight on growth chart	28 (5.5%)	154 (6.2%)	178 (10.1%)	179 (5.8%)	190 (4.2%)	729 (5.9%)
Provider took temperature	308 (60.7%)	2334 (93.2%)	1562 (88.7%)	2131 (69.5%)	2801 (61.7%)	9136 (73.8%)
Provider checked palms / conjunctiva / mouth for pallor	112 (22.1%)	1911 (76.3%)	909 (51.6%)	1429 (46.6%)	2052 (45.2%)	6413 (51.8%)
Provider checked for oedema	27 (5.3%)	235 (9.4%)	101 (5.7%)	266 (8.7%)	334 (7.4%)	963 (7.8%)
Provider checked vaccination card or vaccinated	54 (10.7%)	551 (22.0%)	475 (27.0%)	2412 (78.7%)	1716 (37.8%)	5208 (42.1%)
Provider asked if child received vitamin A within past 6months	4 (0.8%)	146 (5.8%)	41 (2.3%)	71 (2.3%)	174 (3.8%)	436 (3.5%)
Provider asked if child received any deworming medication in past 6 months	5 (1.0%)	303 (12.1%)	54 (3.1%)	69 (2.3%)	266 (5.9%)	697 (5.6%)
Provider explained dosing if medication prescribed	225 (45.6%)	1220 (49.9%)	1043 (65.8%)	1597 (52.6%)	1686 (38.0%)	5771 (48.1%)
Provider recommended food / liquid intake	174 (34.3%)	680 (27.2%)	694 (39.4%)	581 (18.9%)	1190 (26.2%)	3319 (26.8%)
Provider described ≥ 1 danger sign requiring return to facility	44 (8.7%)	289 (11.5%)	90 (5.1%)	379 (12.4%)	770 (17.0%)	1572 (12.7%)
Provider discussed follow-up appointment	26 (5.1%)	340 (13.6%)	628 (35.7%)	744 (24.3%)	1016 (22.4%)	2754 (22.3%)
Provider stated diagnosis to caretaker	178 (35.1%)	846 (33.8%)	266 (15.1%)	1365 (44.5%)	2205 (48.6%)	4860 (39.3%)

For peer review only

Table S3. Items included in the technical quality indicator for children 2 months or younger

	Afghanistan	DRC	Haiti	Malawi	Tanzania	Total
	(N = 49)	(N = 87)	(N = 341)	(N = 109)	(N = 191)	(N = 777)
Provider asked / caretaker mentioned if child unable to drink or breastfeed	15 (30.6%)	39 (43.8%)	97 (29.2%)	52 (46.8%)	74 (38.7%)	277 (35.9%)
Provider asked about normal (breast)feeding pattern	20 (40.8%)	38 (42.7%)	165 (49.7%)	21 (18.9%)	81 (42.4%)	325 (42.1%)
Provider asked about (breast)feeding pattern during this illness	12 (24.5%)	24 (27.0%)	104 (31.3%)	25 (22.5%)	53 (27.7%)	218 (28.2%)
Provider asked / caretaker mentioned diarrhea	19 (38.8%)	24 (27.0%)	49 (14.8%)	28 (25.2%)	89 (46.6%)	209 (27.1%)
Provider asked / caretaker mentioned convulsions	4 (8.2%)	10 (11.2%)	8 (2.4%)	7 (6.3%)	40 (20.9%)	69 (8.9%)
Provider asked about mother's HIV status	0 (0.0%)	1 (1.1%)	17 (5.1%)	9 (8.1%)	24 (12.6%)	51 (6.6%)
Provider counted respiration for 60 seconds	8 (16.3%)	37 (41.6%)	82 (24.7%)	20 (18.0%)	29 (15.2%)	176 (22.8%)
Provider weighed client	35 (71.4%)	66 (74.2%)	273 (82.2%)	24 (21.6%)	33 (17.3%)	431 (55.8%)
Provider plotted weight on growth chart	2 (4.1%)	3 (3.4%)	39 (11.7%)	4 (3.6%)	12 (6.3%)	60 (7.8%)
Provider took temperature	33 (67.3%)	75 (84.3%)	280 (84.3%)	81 (73.0%)	116 (60.7%)	585 (75.8%)
Provider looked into child's mouth	25 (51.0%)	13 (14.6%)	41 (12.3%)	10 (9.0%)	29 (15.2%)	118 (15.3%)
Provider checked vaccination card or vaccinated	8 (16.3%)	32 (36.0%)	124 (37.3%)	97 (87.4%)	86 (45.0%)	347 (44.9%)
Provider asked if child received vitamin A within past 6 months	0 (0.0%)	4 (4.5%)	3 (0.9%)	2 (1.8%)	3 (1.6%)	12 (1.6%)
Provider explained dosing if medication prescribed	21 (53.8%)	43 (51.2%)	165 (59.4%)	53 (50.0%)	48 (27.3%)	330 (48.3%)
Provider recommended food / liquid intake	27 (55.1%)	31 (34.8%)	190 (57.2%)	48 (43.2%)	55 (28.8%)	351 (45.5%)
Provider described ≥ 1 danger sign requiring return to facility	6 (12.2%)	9 (10.1%)	23 (6.9%)	20 (18.0%)	33 (17.3%)	91 (11.8%)
Provider discussed follow-up appt (sick child)	3(6.1%)	15 (16.9%)	115 (34.6%)	24 (21.6%)	44 (23.0%)	201 (26.0%)

For peer review only

Table S4. Modeling strategy for analyses

Model #	Description	Variables included
Model 0 A	Empty model no random intercept	
Model 0 B	Empty model with random intercept	
Model 1	Child and caretaker characteristics	Number of symptoms caretaker identified<br>Child gender<br>Age of child<br>Age of caretaker<br>Education level of caretaker
Model 2	Model 1 + health system foundations	Facility type<br>Facility management<br>Readiness of facility to provide child health services<br>Urban setting<br>Provider type<br>Provider gender<br>Provider years since graduation<br>Provider receives regular salary supplement
Model 3	Model 2 + Process of care	Technical quality of care observed<br>Problem with days or hours services are provided<br>Problem with wait time<br>Problem with cleanliness of facility<br>Problem with how staff treated patient<br>Problem with ability to discuss concerns with provider<br>Problem with amount of explanation provided<br>Problem with medication<br>Problem with cost of services
Model 4	Model 3 + Outer context	Countries

For peer review only

Table S5. Final multilevel models for each country

	Afghanistan				DRC				Haiti				Malawi				Tanzania			
	OR		95% CI		OR		95% C.I.		OR		95% C.I.		OR		95% C. I.		OR		95% C.I.	
Child and caretaker characteristics																				
Number of symptoms caregiver identified	1.0	ns	0.9	1.3	0.8	***	0.7	1.0	1.0	ns	0.9	1.1	0.9	ns	0.8	1.0	1.0	ns	0.9	1.1
Child is female (*versus* male)	0.6	ns	0.3	1.1	0.9	ns	0.6	1.4	1.1	ns	0.8	1.5	0.9	ns	0.6	1.3	1.1	ns	0.8	1.4
Age of child (*versus* 12 months or less)																				
13 months or older	1.3	ns	0.6	2.5	1.1	ns	0.7	1.8	0.8	ns	0.6	1.2	0.8	ns	0.6	1.1	1.1	ns	0.9	1.4
not specified	0.3	*	0.1	0.9	0.3	ns	0.1	1.2	1.0				0.8	ns	0.1	4.5	1.1	ns	0.0	41.2
Age of caretaker (*versus* 20-35 yrs old)																				
0-19 yrs old	2.5	ns	0.3	21.2	1.4	ns	0.7	3.2	0.8	ns	0.4	1.5	1.1	ns	0.7	1.6	1.5	ns	1.0	2.2
36+ yrs old	1.3	ns	0.7	2.5	1.3	ns	0.8	2.3	1.1	ns	0.7	1.7	1.4	ns	0.9	2.4	0.8	ns	0.6	1.2
not specified	1.3	ns	0.5	3.9	0.3	***	0.1	1.0	0.5	ns	0.1	2.0	1.0	ns	0.5	2.3	1.0	ns	0.3	3.4
Education level of caretaker (*versus* none)																				
Primary	0.7	ns	0.2	1.9	0.6	ns	0.3	1.2	1.0	ns	0.4	2.4	0.9	ns	0.6	1.4	0.9	ns	0.6	1.3
Secondary	0.6	ns	0.3	1.5	0.4	***	0.2	0.9	0.8	ns	0.4	1.6	0.7	ns	0.4	1.3	0.7	ns	0.5	1.1
Tertiary or higher	0.3	*	0.1	1.0	0.2	***	0.1	0.7	0.5	ns	0.2	1.0	0.9	ns	0.4	2.0	0.4	*	0.2	0.8
Health system foundations																				
Facility characteristics																				
Types (*versus* hospitals)																				
Health centers	1.6	ns	0.6	4.8	2.2	***	1.3	3.8	1.5	ns	0.9	2.4	0.9	ns	0.6	1.4	1.3	ns	0.9	1.7
Health posts and dispensaries	-								1.8	ns	1.0	3.5	1.4	ns	0.6	3.3	1.7	*	1.1	2.6
Facility management (*versus* public)																				
Private not-for-profit	1.9	ns	0.4	9.8	1.1	ns	0.7	1.9	1.2	ns	0.8	1.9	0.8	ns	0.5	1.2	1.0	ns	0.7	1.4
Private for-profit	0.6	ns	0.2	2.1	2.8	***	1.2	6.2	1.2	ns	0.7	1.9	0.8	ns	0.5	1.4	1.3	ns	0.7	2.4
Readiness of facility to provide child services	1.7	***	1.3	2.1	1.1	ns	1.0	1.3	1.0	ns	0.9	1.1	1.0	ns	0.9	1.1	1.1	ns	1.0	1.2
Urban facility (*versus* rural)	1.4	ns	0.6	3.6	0.8	ns	0.4	1.4	0.5	***	0.3	0.7	0.8	ns	0.5	1.3	0.9	ns	0.6	1.1
Provider characteristics																				
Types (*versus* doctor, advanced pract. clinician)	1.0																			
Nurse, midwife	-				0.7	ns	0.4	1.2	0.6	ns	0.4	1.1	0.9	ns	0.5	1.3	1.1	ns	0.8	1.6
Other - Pharm, lab, dental, non-clinical	-				0.7	ns	0.2	2.5	-				-				0.7	ns	0.4	1.3
Provider is female (*versus* male)	1.8	ns	0.2	20.9	1.0	ns	0.5	2.0	0.8	ns	0.5	1.2	1.2	ns	0.8	1.9	1.0	ns	0.8	1.3
Years since graduation or qualification	1.0	ns	0.9	1.1	1.0	ns	1.0	1.0	1.0	ns	1.0	1.0	1.0	ns	1.0	1.0	1.0	ns	1.0	1.0
Regular salary supplements for provider (*vs* none)	1.2	ns	0.5	3.0	0.7	ns	0.3	1.5	1.3	ns	0.7	2.3	1.0	ns	0.7	1.4	1.1	ns	0.8	1.4
Process of care																				
Technical quality of care observed	0.8	ns	0.6	1.1	1.0	ns	0.8	1.1	1.2	*	1.0	1.4	1.0	ns	0.9	1.1	1.2	***	1.1	1.3
Problem with days or hours services are provided	1.0	ns	0.4	2.7	1.5	ns	0.7	3.3	0.6	ns	0.3	1.1	0.8	ns	0.5	1.2	1.0	ns	0.7	1.5
Problem with wait time	0.5	ns	0.2	1.1	0.5	ns	0.3	1.0	0.3	***	0.2	0.4	0.7	*	0.5	1.0	0.6	***	0.4	0.7
Problem with cleanliness of facility	0.7	ns	0.2	2.2	0.9	ns	0.5	1.9	0.5	*	0.2	1.0	0.7	ns	0.4	1.0	0.6	**	0.4	0.8

For peer review only

Problem with how staff treated patient	0.2	***	0.1	0.5	0.8	ns	0.3	2.1	0.2	***	0.1	0.5	0.4	***	0.2	0.6	0.6	ns	0.4	1.0
Problem with ability to discuss concerns with provider	0.3	**	0.1	0.9	2.2	ns	0.7	6.5	1.6	ns	0.8	2.9	0.6	ns	0.3	1.2	0.6	*	0.4	1.0
Problem with amount of explanation provided	0.2	***	0.1	0.5	0.7	ns	0.3	1.9	0.4	**	0.2	0.8	0.6	ns	0.3	1.2	0.4	***	0.2	0.7
Problem with medication availability	0.4	**	0.2	0.8	0.2	***	0.1	0.3	0.5	**	0.3	0.8	0.5	***	0.3	0.7	0.2	***	0.1	0.2
Problem with cost of services (None)																				
Problem with cost	0.2	***	0.1	0.5	0.5	***	0.3	0.8	0.5	**	0.2	0.9	0.6	*	0.3	1.0	0.5	***	0.3	0.8
Not specified	-				0.9	ns	0.3	2.9	-				1		0.4	2.4	1.21		0.2	9.2

* $p \leq 0.05$; ** $p \leq 0.01$; *** $p \leq 0.001$

Numbers were rounded to 1 decimal place for space

For peer review only

STROBE Statement—Checklist of items that should be included in reports of ***cross-sectional studies***

	Item No	Recommendation	Page No
**Title and abstract**	1	(*a*) Indicate the study's design with a commonly used term in the title or the abstract	1
		(*b*) Provide in the abstract an informative and balanced summary of what was done and what was found	1
**Introduction**			
Background/rationale	2	Explain the scientific background and rationale for the investigation being reported	3-4
Objectives	3	State specific objectives, including any prespecified hypotheses	4
**Methods**			
Study design	4	Present key elements of study design early in the paper	4
Setting	5	Describe the setting, locations, and relevant dates, including periods of recruitment, exposure, follow-up, and data collection	4
Participants	6	(*a*) Give the eligibility criteria, and the sources and methods of selection of participants	4-5
Variables	7	Clearly define all outcomes, exposures, predictors, potential confounders, and effect modifiers. Give diagnostic criteria, if applicable	5-7
Data sources/ measurement	8*	For each variable of interest, give sources of data and details of methods of assessment (measurement). Describe comparability of assessment methods if there is more than one group	5-7
Bias	9	Describe any efforts to address potential sources of bias	7
Study size	10	Explain how the study size was arrived at	4
Quantitative variables	11	Explain how quantitative variables were handled in the analyses. If applicable, describe which groupings were chosen and why	7
Statistical methods	12	(*a*) Describe all statistical methods, including those used to control for confounding	7
		(*b*) Describe any methods used to examine subgroups and interactions	7
		(*c*) Explain how missing data were addressed	9
		(*d*) If applicable, describe analytical methods taking account of sampling strategy	7
		(*e*) Describe any sensitivity analyses	N/A
**Results**			
Participants	13*	(a) Report numbers of individuals at each stage of study—eg numbers potentially eligible, examined for eligibility, confirmed eligible, included in the study, completing follow-up, and analysed	9
		(b) Give reasons for non-participation at each stage	N/A
		(c) Consider use of a flow diagram	N/A

For peer review only - http://bmjopen.bmj.com/site/about/guidelines.xhtml

Descriptive data	14*	(a) Give characteristics of study participants (eg demographic, clinical, social) and information on exposures and potential confounders	9-10
		(b) Indicate number of participants with missing data for each variable of interest	9
Outcome data	15*	Report numbers of outcome events or summary measures	Table 2, page 9-10
Main results	16	(*a*) Give unadjusted estimates and, if applicable, confounder-adjusted estimates and their precision (eg, 95% confidence interval). Make clear which confounders were adjusted for and why they were included	10-12
		(*b*) Report category boundaries when continuous variables were categorized	5-6
		(*c*) If relevant, consider translating estimates of relative risk into absolute risk for a meaningful time period	N/A
Other analyses	17	Report other analyses done—eg analyses of subgroups and interactions, and sensitivity analyses	13-14 and supplementary file
**Discussion**			
Key results	18	Summarise key results with reference to study objectives	15
Limitations	19	Discuss limitations of the study, taking into account sources of potential bias or imprecision. Discuss both direction and magnitude of any potential bias	16-17
Interpretation	20	Give a cautious overall interpretation of results considering objectives, limitations, multiplicity of analyses, results from similar studies, and other relevant evidence	15-17
Generalisability	21	Discuss the generalisability (external validity) of the study results	16
**Other information**			
Funding	22	Give the source of funding and the role of the funders for the present study and, if applicable, for the original study on which the present article is based	See cover page

*Give information separately for exposed and unexposed groups.

Note: An Explanation and Elaboration article discusses each checklist item and gives methodological background and published examples of transparent reporting. The STROBE checklist is best used in conjunction with this article (freely available on the Web sites of PLoS Medicine at http://www.plosmedicine.org/, Annals of Internal Medicine at http://www.annals.org/, and Epidemiology at http://www.epidem.com/). Information on the STROBE Initiative is available at www.strobe-statement.org.

For peer review only

For peer review only - http://bmjopen.bmj.com/site/about/guidelines.xhtml

BMJ Open

What are the determinants of variation in caretaker satisfaction with sick child consultations? a cross-sectional analysis in five low- and middle-income countries

Journal:	*BMJ Open*
Manuscript ID	bmjopen-2022-071037.R1
Article Type:	Original research
Date Submitted by the Author:	08-May-2023
Complete List of Authors:	Turcotte-Tremblay, Anne-Marie; Université Laval; Harvard University T H Chan School of Public Health, Global Health and Population<br>Lee, Hwa-Young; Harvard University T H Chan School of Public Health<br>Kruk, Margaret; Harvard University T H Chan School of Public Health, Global Health and Population
**Primary Subject Heading**:	Health services research
Secondary Subject Heading:	Patient-centred medicine
Keywords:	Quality in health care < HEALTH SERVICES ADMINISTRATION & MANAGEMENT, Community child health < PAEDIATRICS, HEALTH SERVICES ADMINISTRATION & MANAGEMENT

SCHOLARONE™
Manuscripts

I, the Submitting Author has the right to grant and does grant on behalf of all authors of the Work (as defined in the below author licence), an exclusive licence and/or a non-exclusive licence for contributions from authors who are: i) UK Crown employees; ii) where BMJ has agreed a CC-BY licence shall apply, and/or iii) in accordance with the terms applicable for US Federal Government officers or employees acting as part of their official duties; on a worldwide, perpetual, irrevocable, royalty-free basis to BMJ Publishing Group Ltd ("BMJ") its licensees and where the relevant Journal is co-owned by BMJ to the co-owners of the Journal, to publish the Work in this journal and any other BMJ products and to exploit all rights, as set out in our licence.

The Submitting Author accepts and understands that any supply made under these terms is made by BMJ to the Submitting Author unless you are acting as an employee on behalf of your employer or a postgraduate student of an affiliated institution which is paying any applicable article publishing charge ("APC") for Open Access articles. Where the Submitting Author wishes to make the Work available on an Open Access basis (and intends to pay the relevant APC), the terms of reuse of such Open Access shall be governed by a Creative Commons licence – details of these licences and which Creative Commons licence will apply to this Work are set out in our licence referred to above.

Other than as permitted in any relevant BMJ Author's Self Archiving Policies, I confirm this Work has not been accepted for publication elsewhere, is not being considered for publication elsewhere and does not duplicate material already published. I confirm all authors consent to publication of this Work and authorise the granting of this licence.

For peer review only

What are the determinants of variation in caretaker satisfaction with sick child consultations? a cross-sectional analysis in five low- and middle-income countries

Authors: Turcotte-Tremblay, Anne-Marie[1,2], Lee, Hwa-Young[2,3]*, Kruk, Margaret E.[2]

Affiliations:

1. Faculty of Nursing, Université Laval, Laval, QC, Canada
2. Department of Global Health and Population, Harvard T.H. Chan School of Public Health, Boston, MA, United States
3. Graduate School of Public Health and Healthcare Management, The Catholic University of Korea

*Corresponding author:

Hwa-Young Lee

Graduate School of Public Health and Healthcare Management, The Catholic University of Korea. 222 Banpo-daero Seocho-gu, Seoul, (06591) Republic of Korea
diana0224@gmail.com

Telephone: 82-20-2230-4923

Word count: 3 900

Keywords: satisfaction, process of care, variance explained, multilevel modeling, low- and middle-income countries

For peer review only - http://bmjopen.bmj.com/site/about/guidelines.xhtml

ABSTRACT

Objectives: The objective of this study was to explore determinants of variation in overall caretaker satisfaction with curative care for sick children under the age of five in five low-and middle-income countries.

Design: A pooled cross-sectional analysis was conducted using data from the Service Provision Assessment.

Setting: We used data collected in five countries (Afghanistan, Democratic Republic of the Congo, Haiti, Malawi, and Tanzania) between 2013–2018.

Participants: Respondents were 13,149 caretakers of children under the age of five who consulted for a sick child visit.

Primary outcomes measured: The outcome variable was whether the child's caretaker was very satisfied versus more or less satisfied or not satisfied overall. Predictors pertained to child and caretaker characteristics, health system foundations, and process of care (e.g., care competence, user experience). Two-level logistic regression models were used to assess the extent to which these categories of variables explained variation in satisfaction. The main analyses used pooled data; country-level analyses were also performed.

Results**:** Process of care, including user experience, explained the largest proportion of variance in caretaker satisfaction (13.8%), compared to child and caretaker characteristics (0.9%) and health system foundations (3.8%). The odds of being very satisfied were lower for caretakers who were not given adequate explanation (OR: 0.56, 95% CI: 0.46 to 0.67), who had a problem with medication availability (OR: 0.31, 95% CI: 0.27 to 0.35) or who encountered a problem with the cost of services (OR: 0.57, 95% CI: 0.48 to 0.66). The final model explained only 21.8% of the total variance. Country level analyses showed differences in variance explained and in associations with predictors.

Conclusions: Better process of care, especially user experience, should be prioritized for its benefit regarding caretaker satisfaction. Unmeasured factors explained the majority of variation in caretaker satisfaction and should be explored in future studies.

For peer review only - http://bmjopen.bmj.com/site/about/guidelines.xhtml

Keywords: satisfaction, process of care, variance explained, multilevel modeling, low- and middle-income countries

Strengths and limitations of this study

- This is the first multi-country analysis to examine the determinants of variation in caretaker satisfaction with healthcare services for sick children under the age of five in LMICs.
- The study has the advantage of combining multiple sources of data (facility audits, health provider interviews and exit interviews with caretakers).
- Novel evidence shows that the process of care, including the technical quality of care and the caretaker experience, explains the largest proportion of variance in caretaker satisfaction with sick childcare across five LMICs.
- The satisfaction rates reported by caretakers were generally high, possibly due to desirability bias in participants' responses during interviews, low patient expectations, and behaviors during the audits.

For peer review only - http://bmjopen.bmj.com/site/about/guidelines.xhtml

BACKGROUND

User satisfaction is an important dimension of high-quality health systems [1]. It reflects the ultimate judgment of the consumer and influences decisions on when and where to seek care [1]. Consequently, user satisfaction measures are increasingly used to evaluate the performance of healthcare systems, improve accountability, and provide financial incentives to healthcare staff. The results of user satisfaction surveys are also used to help managers and healthcare providers identify service factors needing improvement [2].

However, whether and to what extent user satisfaction measures are appropriate to evaluate health systems depends on the extent to which they are influenced by health system versus other factors. Studies have shown that user satisfaction is a complex and multidimensional concept with numerous determining factors. Batbaatar *et al* [2] reported that user satisfaction is influenced by technical care, interpersonal care, physical environment, access, organizational characteristics, continuity of care, and outcome of care. Focusing on child health, McCormick *et al* [3] identified the health status of the child, sociodemographic characteristics, and history of treatment as predictors of parental satisfaction in the United States (US). However, these analyses have not paid much attention to broader factors (e.g., facility characteristics, country-level characteristics) that may influence user satisfaction.

Understanding the relative contributions of different types of factors to explaining variation in user satisfaction is useful to better interpret and use results from user satisfaction surveys for quality improvement. However, few studies have examined how much variation is explained by determinants of user satisfaction in various countries. One study used data from 21 European Union countries to examine variation in user satisfaction with the health system [4]. Those authors found that all factors taken into account in their model, such as patient expectations, health status, and personality, explained only 17.5% of the observed variation in user satisfaction, of which 10.4% was explained by user experience with system responsiveness [4]. They concluded that, contrary to published reports, user experience accounts for only a small fraction of variation in user satisfaction with the healthcare system. However, the proportion of variance in user satisfaction explained by health system and non-health system factors in low- and middle-income countries (LMICs) remains unclear. Most evidence on this issue was derived in high-income countries.

For peer review only - http://bmjopen.bmj.com/site/about/guidelines.xhtml

The objectives of our study were twofold: 1) to assess the proportion of variance in caretaker overall satisfaction explained by factors related to child and caretaker characteristics, health system foundations, and process of care; 2) to identify variables associated with caretaker overall satisfaction in five LMICs.

METHODS

Study setting

Table 1 presents the demographic characteristics and healthcare situations in the five countries included in this study. All of these countries are characterized by high rates of neonatal mortality. DRC had the highest mortality rate for children under the age of five per 1,000 live births (58) and the lowest health spending per capita (51 PPP$). In contrast, Malawi had the lowest mortality rate for children under the age of five (39) and the second lowest value for health spending per capita (82 PPP$).

Table 1. Context of study countries

	Afghanistan	DRC	Haiti	Malawi	Tanzania
Population (in millions)*	38.0	86.8	11.3	18.6	58.0
Urban population (% of total population)†	26	46	57	17	35
Gross domestic product per person (USD)*	507	581	1,273	412	1,122
Health expenditure per capita (PPP$)*	286	51	143	82	99
Primary education completion rate, total (% of relevant group)‡	84	70	49	80	69
Literacy rate of adults (%) ‡§	43	77	62	62	78
Birth attended by skilled health staff (%)‡	59	80	42	90	64
Maternal mortality (per 100,000 live births)**	638	473	480	349	524
Neonatal mortality (per 1,000 live births)*	36	27	25	20	20
Mortality rate under age five (per 1,000 live births)†	58	81	61	39	49
Hospital beds (per 1,000 people)‡	0.4	0.8	0.7	1.3	0.7

* Data from 2019. † Data from 2020. ‡ Data from the most recent year available.

§ Percentage of people aged 15 and above able to read & write simple statements about everyday life.

** Data from 2017.

For peer review only - http://bmjopen.bmj.com/site/about/guidelines.xhtml

Data source and sampling

The data were obtained from Service Provision Assessment (SPA) surveys. SPA surveys are conducted by the Demographic and Health Survey (DHS) Program of United States Agency for International Development (USAID), with a national statistics agency in the countries surveyed to measure the capacity of health systems in LMICs. The datasets are available online [5–9]. For the SPA survey, facilities are selected from a comprehensive list of health facilities in each country, categorized by facility type, managing authority, and region; a nationally representative sample of health facilities is selected. A description of the methodology used for SPA surveys is available online [10].

We limited our study population to the most recent SPA surveys that included a question on user satisfaction with consultation for sick children under the age of five in five countries: Afghanistan (2018), Democratic Republic of the Congo (DRC) (2018), Haiti (2017), Malawi (2013), and Tanzania (2015).

All caretakers who sought care for a sick child consultation in hospitals or primary health care facilities were included in our study without any specific exclusion criteria. We used data from four separate modules: facility audits, health provider interviews, observation of protocols for sick child care, and exit interviews with caretakers. Facility audits collected data on available human resources, basic amenities, and availability of essential equipment, supplies, and medicines. A sample of health providers was selected from the facility roster to be interviewed and observed. Trained enumerators observed clinical visits to assess adherence to clinical guidelines during consultations for sick children. The caretakers (e.g., parents, grandparents) of children observed during the consultations completed an exit interview regarding their level of satisfaction with the services received, problems encountered, and demographic characteristics.

Data management and quality control

The procedures for data management and quality were similar across countries [11–15]. The interviewers were supposed to review the data and enter it in tablets. The data files were transferred to a supervisor who oversaw the data collection process. When supervisors noted missing information or errors, they sent the data back to the interviewer for revision. Then, the data was sent to a central office via the Internet. In the central office, data processors detected inconsistencies and gave feedback to the team in the field to resolve the problems. For tracking of

For peer review only - http://bmjopen.bmj.com/site/about/guidelines.xhtml

systematic errors arising from each interviewer, field check tables were run. If an interviewer committed errors systematically, the central office and coordinators followed up with the interviewers [11–15].

Framework

To conceptualize our analyses, we adapted the High Quality Health Systems (HQHS) framework from the Lancet Global Health Commission [1]. As shown in Figure 1, this framework comprises three key domains: foundations, process of care, and quality impacts [1]. Health system foundations include the population and their health needs, health sector governance, care delivery platforms, workforce skills, and tools such as medicines and equipment. Process of care is composed of two sub-domains: competent care and user experience. Competent care refers to systematic assessment, correct diagnosis, appropriate treatment, counseling, prevention, and detection. Positive user experience refers to respect, autonomy, clear communication, short wait times, patient affordability, and ease of use. Caretaker satisfaction, our outcome of interest, is a quality impact measure that contributes to confidence in the system and willingness to use health care.

INSERT Figure 1. Conceptual framework

Outcome variable

The outcome variable is caretaker satisfaction, extracted from the exit interview module. The following question was used to elicit caretaker satisfaction regarding the sick child consultation: "In general, which of the following statements best describes your opinion of the services you either received or were provided at this facility today? Very satisfied, more or less satisfied, or not satisfied." Based on the low levels of dissatisfaction, we created a binary variable of satisfaction equal to 1 if the caretaker was very satisfied and 0 otherwise (i.e., if the caretaker was more or less satisfied, or not satisfied).

Predictor variables

We identified child and caretaker characteristics that were likely to influence user satisfaction by reviewing previous studies [1–4]. Child characteristics included gender (male or female), age (12 months or less, 13 months to 59 months, and age not specified), and severity of symptoms, constructed by summing the reasons the caretaker identified for bringing in the child, including

For peer review only - http://bmjopen.bmj.com/site/about/guidelines.xhtml

cough, diarrhea, fever, vomiting, problem with feeding, convulsions, excessive sleepiness, or other symptoms (range: 1~8). Caretaker characteristics included age (≤19, 20–35, ≥36 years, and age not specified) and education level (none, primary, secondary, tertiary, or higher).

To characterize the facilities, we examined the types of management authorities (public, private not-for-profit, private for-profit) and whether providers received a regular salary supplement. Facility types categorized into hospitals, health centers or clinics, health posts or dispensaries, and urban setting (versus rural) were included. To characterize the workforce, providers were separated into three categories: physicians/clinical officers/assistant medical officers; nurses/midwives; and aides/assistants. Other provider characteristics included gender and years since graduation (as a proxy for experience). To characterize the facilities' tools, we drew on data from facility audits to calculate a service readiness score for preventive and curative care regarding sick child health based on the World Health Organization (WHO) Service Availability and Readiness Assessment (SARA) guidelines [16]. The overall score was calculated by averaging all items in four domains: staff and training, equipment, diagnostics, and medicines and commodities. The original scores ranged between 0 and 1 but were multiplied by 10 to facilitate interpretation (0–10). Table S1 presents the full list of indicators used (see supplementary file).

We assessed the technical quality of sick child consultations by investigating the degree to which the provider adhered to clinical guidelines during the consultation observation [17,18]. The technical quality score was calculated as the percentage of clinically recommended tasks performed in four domains: patient history-taking, physical exam, drug administration and immunization, and client education and counseling. We also multiplied the score by 10 to facilitate interpretation. The items included for technical quality indicators for sick child care are presented in the supplementary file (Table S2 and Table S3).

Caretakers' perceptions of their experience during the visit were examined through exit interviews by asking whether they had encountered problems that day related to: 1) days or hours of service at this facility (i.e., when it opened and closed); 2) wait time to see a provider; 3) facility cleanliness; 4) how staff treated them; 5) ability to discuss problems or concerns about the health issue; 6) amount of explanation received about the problem or treatment; 7) medication; and 8) cost of services or treatments. For each of these, responses were scored as the number of problems encountered. We included the country variable as a proxy for country-level outer context.

For peer review only - http://bmjopen.bmj.com/site/about/guidelines.xhtml

Statistical analysis

We performed a complete case analysis, which yielded a total of 13,149 sick child visits across 3,421 facilities for the sample in the five countries. For variables with the most missing data (e.g., child age, caretaker age), we added the value "not specified" to be able to include the cases in the analysis. Less than 5% of the sample had missing data, in total.

We calculated descriptive statistics for all variables of interest, presenting the proportions for categorical variables and mean and standard deviation (SD) for continuous covariates. These descriptive analyses incorporated survey sampling weights for patients and facilities.

For the main analyses, we performed a two-level logistic regression analysis based on pooled data from the five countries, where level 1 was the consultation and level 2 was the facility. This provided variance estimates while accounting for clustering effect at the facility level. The following general structure was used:

$$logit\ (\Pr\ (Y_{ji} = 1|X) = \beta_0 + \beta \cdot X_{ij} + u_{0j}$$

where Y (caretaker satisfaction) and X (representing a vector of independent variables) were each assumed to follow a two-level data structure, with both fixed-effects (β_0,$\beta \cdot X_{ij}$) and random-effects parameters (u_{0j}). The random-effects parameter (u_{0j}) was assumed to follow a normal distribution, with mean of 0 and variances of u_{0j} ~ N (0, $\sigma^2_{u_0}$). We confirmed the linearity between the four continuous independent variables and log-odds of the outcome using the Box-Tidwell test.

Four models were estimated based on the general modeling structure outlined above, by incrementally adding blocks of covariates following our conceptual framework (see Table S4 in the supplementary file for details on the modeling strategy). M0 was a null/unadjusted model with an intercept term in the fixed part of the model. M1 added child and caretaker characteristics. M2 built on the previous model to include variables related to health system foundations. M3 added

For peer review only - http://bmjopen.bmj.com/site/about/guidelines.xhtml

variables related to the process of care. For the analyses with the pooled data, M4 included the country as a variable.

We applied Snijders and Bosker's method to measure the explained proportion of variance in caretaker satisfaction for the logistic multilevel model (details are provided in Box 1 of the Supplementary file) [19]. Since logistic regression models do not have a level 1 residual term, unexplained variance at the consultation level was estimated as $\pi^2/3 \approx 3.29$ based on the method summarized by Goldstein *et al* (8). When considering only the unexplained part of the total variance, the proportion attributed to the health facilities level (called residual intraclass correlation) was calculated.

We also performed country-stratified analyses to examine whether the results were country-specific. All analyses were conducted in Stata 16.0 (StataCorp, College Station, Texas).

Patient and public involvement statement

Patients and/or the public were not involved in the design, or conduct, or reporting, or dissemination of this research.

Ethical consideration

SPA data are publicly accessible from the DHS website. Participants provided consent for the original data collection. This secondary analysis of deidentified data was deemed exempt from human subjects review.

RESULTS

Descriptive statistics

Table 2 presents summary statistics for the sample. Overall, 77% of caretakers were very satisfied. The percentage of very satisfied respondents was highest in Haiti (89%) and lowest in Afghanistan (62%). Approximately 68% of caretakers had primary education level or less. Of the providers,

For peer review only - http://bmjopen.bmj.com/site/about/guidelines.xhtml

34% were female, and 58% were qualified as doctors or advance practice clinicians. The majority of facilities were health centers (52%) and public (63%). The mean scores for technical quality of care observed were quite low (3.1). Because data in Afghanistan only cover urban areas, the percentage of highly qualified providers, hospitals, and private for-profit facilities was notably higher than in the other countries (Table 2).

For peer review only

For peer review only - http://bmjopen.bmj.com/site/about/guidelines.xhtml

Table 2. Descriptive statistics per country

Variables	Afghanistan N (%)	DRC N (%)	Haiti N (%)	Malawi N (%)	Tanzania N (%)	Total N (%)
Very satisfied/total analytic sample, N (very satisfied, %)	345/556 (62)	1859/2592 (71)	1850/2093 (89)	2581/3177 (82)	3538/4731 (75)	10171/13149 (77)
Child and caretaker characteristics						
Number of symptoms caretaker identified (0-8), Mean (SD)	3.2 (1.3)	3.4 (1.4)	2.9 (1.4)	2.5 (1.2)	2.7 (1.3)	2.8 (1.3)
Child is female	236 (43%)	1238 (47%)	974 (47%)	1566 (50%)	2333 (49%)	6346 (48%)
Age of child (months)						
12 or less	263 (47%)	959 (37%)	1026 (49%)	1300 (41%)	1974 (42%)	5520 (42%)
13 to 59 months	246 (44%)	1618 (62%)	1045 (50%)	1833 (58%)	2768 (58%)	7508 (57%)
not specified	47 (8%)	33 (1%)	15 (1%)	21 (1%)	8 (0%)	123 (1%)
Age of caretaker (yrs)						
20-35	366 (66%)	1799 (69%)	1489 (71%)	2471 (78%)	3505 (74%)	9628 (73%)
≥19	19 (3%)	222 (8%)	166 (8%)	324 (10%)	439 (9%)	1167 (9%)
36+	110 (20%)	424 (16%)	407 (20%)	249 (8%)	719 (15%)	1906 (14%)
not specified	62 (11%)	167 (6%)	24 (1%)	111 (3%)	87 (2%)	449 (3%)
Education of caretaker						
None	308 (55%)	569 (22%)	231 (11%)	385 (12%)	901 (19%)	2392 (18%)
Primary	53 (9%)	828 (32%)	583 (28%)	2021 (64%)	3072 (65%)	6556 (50%)
Secondary	121 (22%)	1065 (41%)	1105 (53%)	683 (22%)	693 (15%)	3665 (28%)
Tertiary or higher	74 (13%)	149 (6%)	167 (8%)	66 (2%)	83 (2%)	538 (4%)
Health system foundations						
Provider is female	14 (3%)	576 (14%)	991 (60%)	568 (29%)	2288 (45%)	4435 (34%)
Provider type						
Doctor, advanced practice clinician, paramedical	460 (100%)	1225 (31%)	1196 (72%)	1631 (83%)	3112 (61%)	7622 (58%)
Nurse, midwife	0 (0%)	2689 (67%)	463 (28%)	324 (16%)	1938 (38%)	5413 (41%)
Others - Pharm, lab, dental, non-clinical	0 (0%)	90 (2%)	4 (0%)	11 (1%)	12 (0%)	115 (1%)
Provider receives regular salary supplements	282 (61%)	579 (14%)	168 (10%)	746 (38%)	1461 (29%)	3234 (25%)
Service readiness score for sick child care (0-10), Mean (SD)	6.0 (1.3)	6.5 (1.8)	5.7 (1.6)	6.0 (1.6)	5.2 (1.6)	5.8 (1.7)
Facility type						
Hospitals	353 (61%)	520 (22%)	558 (23%)	591 (16%)	288 (7%)	2308 (18%)
Health centers	227 (39%)	1861 (78%)	1283 (53%)	2804 (78%)	627 (15%)	6800 (52%)
Health posts and dispensaries	0 (0%)	0 (0%)	563 (23%)	195 (5%)	3285 (78%)	4042 (31%)
Facility management						
Public	157 (27%)	1506 (63%)	800 (33%)	2334 (65%)	3486 (83%)	8281 (63%)
Private not-for-profit	71 (12%)	576 (24%)	906 (38%)	748 (21%)	383 (9%)	2682 (20%)
Private for-profit	352 (61%)	299 (13%)	697 (29%)	509 (14%)	332 (8%)	2187 (17%)
Urban facility	575 (99%)	446 (19%)	1128 (47%)	749 (21%)	1070 (25%)	3966 (30%)
Process of care						
Technical quality of care observed (0-10), mean (SD)	2.4 (1.1)	3.4 (1.2)	3.1 (1.2)	3.0 (1.3)	3.1 (1.5)	3.1 (1.4)

For peer review only

Problem with days or hours services are provided	73 (13%)	326 (12%)	128 (6%)	601 (19%)	612 (13%)	1738 (13%)
Problem with wait time	137 (25%)	530 (20%)	474 (23%)	998 (32%)	1329 (28%)	3466 (26%)
Problem with cleanliness of facility	92 (16%)	481 (18%)	100 (5%)	250 (8%)	769 (16%)	1690 (13%)
Problem with how staff treated patient	100 (18%)	269 (10%)	48 (2%)	229 (7%)	317 (7%)	961 (7%)
Problem with ability to discuss concerns with provider	77 (14%)	323 (12%)	180 (9%)	354 (11%)	397 (8%)	1328 (10%)
Problem with amount of explanation provided	75 (13%)	372 (14%)	163 (8%)	356 (11%)	401 (8%)	1365 (10%)
Problem with medication availability	156 (28%)	581 (22%)	205 (10%)	635 (20%)	1540 (32%)	3115 (24%)
Problem with cost of services	193 (35%)	575 (22%)	105 (5%)	174 (6%)	462 (10%)	1508 (11%)

For peer review only

For peer review only - http://bmjopen.bmj.com/site/about/guidelines.xhtml

Total variance explained

Figure 2 shows the percentage of variance explained in each model. Variance explained in the fully adjusted model with the combined data from five countries was 21.8%. However, it varied significantly across countries, ranging from 9.0% in DRC to 51.7% in Afghanistan. Factors related to patient and caretaker characteristics only explained 0.9% of the total variance (0.8% in Tanzania ~ 4.3% in Afghanistan). The explained variance increased by 3.8% when variables related to health system foundations, such as facility and provider characteristics, were included (0.9% in Malawi ~ 10.8% Afghanistan). The largest percentage of the total variance was explained by process of care in both the pooled and country-stratified analyses. When process of care variables were added, the explained variance increased by 13.8% in the pooled analyses. Variance explained by process of care was notably higher in Afghanistan and Tanzania (36.6% and 24.2%, respectively) than in the other countries (5.8% in DRC ~ 10.4% in Malawi).

INSERT Figure 2. Variance explained with addition of variables to the model

Determinants of caretaker satisfaction

Figure 3 shows the results of the final model's fixed part based on the pooled data from the five countries. More severe symptoms of the child (OR: 0.94, 95% CI: 0.91 to 0.98) and higher education of caretakers (OR: 0.78 and 0.53, 95% CI: 0.66 to 0.93 and 0.40 to 0.69 for secondary and tertiary level or higher, respectively) were associated with lower odds of being very satisfied with the service received. Caretakers who sought care in hospital were less likely to be very satisfied with service received than those at lower-level facilities. In particular, caretakers who visited health posts and dispensaries for their sick child were 1.82 times more likely to be very

For peer review only - http://bmjopen.bmj.com/site/about/guidelines.xhtml

satisfied with the service than those who visited hospitals (OR: 1.33; 95% CI: 1.11 to 1.59 and OR: 1.82; 95% CI: 1.40 to 2.38, respectively). Caretakers whose child received care in facilities with higher service readiness scores were more likely to be very satisfied.

Problems with process of care were strong predictors of user satisfaction. Higher technical quality scores for sick child care increased the likelihood of being very satisfied (OR: 1.08, 95% CI: 1.04 to 1.13 per 10% increase). Negative user experiences significantly decreased the odds of caretaker satisfaction. In particular, caretakers who perceived they were not given adequate explanation and perceived a problem with medication availability were 0.56 and 0.31 times less likely to be very satisfied with the service for their sick child, respectively (95% CI: 0.46 to 0.67 and 0.27 to 0.35, respectively). Problems with the cost of services significantly reduced the odds of caretaker satisfaction (OR: 0.57, 95% CI: 0.48 to 0.66). None of the providers' characteristics were associated with caretaker satisfaction. All country dummy variables, which served as proxies for national context, were significant. Compared to caretakers in Malawi, those in Afghanistan, DRC, and Tanzania were less likely to be very satisfied with services, while those in Haiti were 1.76 times more likely to be very satisfied.

INSERT Figure 3. Determinants of caretaker satisfaction

The results of the fixed country effects are presented in Table 3). Tertiary or higher education was associated with satisfaction in Tanzania (OR: 0.4, 95% 0.2 to 0.8), Afghanistan (OR: 0.3, 95% CI: 0.1 to 1.0) and DRC (OR: 0.2, 95% CI: 0.1 to 0.7). In DCR, some facility characteristics appeared to be important determinants of satisfaction. More specifically, caregivers in health centers or private for-profit facilities had higher odds of being very satisfied compared to

For peer review only - http://bmjopen.bmj.com/site/about/guidelines.xhtml

those seeking care in hospitals or public facilities (OR: 2.2, 95% CI: 1.3 to 3.8; OR: 2.8, 95% CI:1.2 to 6.2, respectively). On the other hand, in DRC some aspects of the patients' experiences, such as wait time (OR: 0.5, 95% CI: 0.3 to 1.0) and how staff treated patients (OR: 0.8, 95% CI: 0.8 0.3 to 2.1) were not associated with caretaker satisfaction. Caregivers who had problems with how the staff treated them had lower odds of being very satisfied in Afghanistan, Haiti and Malawi. In all five countries, caregivers who encountered problems with medication availability and cost of services were significantly less likely to be very satisfied (see Table 3).

For peer review only

For peer review only - http://bmjopen.bmj.com/site/about/guidelines.xhtml

Table 3. Final multilevel models for each country

	Afghanistan				DRC				Haiti				Malawi				Tanzania			
	OR		95% CI		OR		95% C.I.		OR		95% C.I.		OR		95% C. I.		OR		95% C.I.	
Child and caretaker characteristics																				
Number of symptoms caregiver identified	1.0	ns	0.9	1.3	0.8	***	0.7	1.0	1.0	ns	0.9	1.1	0.9	ns	0.8	1.0	1.0	ns	0.9	1.1
Child is female (*versus* male)	0.6	ns	0.3	1.1	0.9	ns	0.6	1.4	1.1	ns	0.8	1.5	0.9	ns	0.6	1.3	1.1	ns	0.8	1.4
Age of child (*versus* 12 months or less)																				
13 to 59 months	1.3	ns	0.6	2.5	1.1	ns	0.7	1.8	0.8	ns	0.6	1.2	0.8	ns	0.6	1.1	1.1	ns	0.9	1.4
not specified	0.3	*	0.1	0.9	0.3	ns	0.1	1.2	1.0				0.8	ns	0.1	4.5	1.1	ns	0.0	41.2
Age of caretaker (*versus* 20-35 yrs old)																				
≥19 yrs old	2.5	ns	0.3	21.2	1.4	ns	0.7	3.2	0.8	ns	0.4	1.5	1.1	ns	0.7	1.6	1.5	ns	1.0	2.2
36+ yrs old	1.3	ns	0.7	2.5	1.3	ns	0.8	2.3	1.1	ns	0.7	1.7	1.4	ns	0.9	2.4	0.8	ns	0.6	1.2
not specified	1.3	ns	0.5	3.9	0.3	***	0.1	1.0	0.5	ns	0.1	2.0	1.0	ns	0.5	2.3	1.0	ns	0.3	3.4
Education level of caretaker (*versus* none)																				
Primary	0.7	ns	0.2	1.9	0.6	ns	0.3	1.2	1.0	ns	0.4	2.4	0.9	ns	0.6	1.4	0.9	ns	0.6	1.3
Secondary	0.6	ns	0.3	1.5	0.4	***	0.2	0.9	0.8	ns	0.4	1.6	0.7	ns	0.4	1.3	0.7	ns	0.5	1.1
Tertiary or higher	0.3	*	0.1	1.0	0.2	***	0.1	0.7	0.5	ns	0.2	1.0	0.9	ns	0.4	2.0	0.4	*	0.2	0.8
Health system foundations																				
Facility characteristics																				
Types (*versus* hospitals)																				
Health centers	1.6	ns	0.6	4.8	2.2	***	1.3	3.8	1.5	ns	0.9	2.4	0.9	ns	0.6	1.4	1.3	ns	0.9	1.7
Health posts and dispensaries	-								1.8	ns	1.0	3.5	1.4	ns	0.6	3.3	1.7	*	1.1	2.6
Facility management (*versus* public)																				
Private not-for-profit	1.9	ns	0.4	9.8	1.1	ns	0.7	1.9	1.2	ns	0.8	1.9	0.8	ns	0.5	1.2	1.0	ns	0.7	1.4
Private for-profit	0.6	ns	0.2	2.1	2.8	***	1.2	6.2	1.2	ns	0.7	1.9	0.8	ns	0.5	1.4	1.3	ns	0.7	2.4
Readiness of facility to provide child services	1.7	***	1.3	2.1	1.1	ns	1.0	1.3	1.0	ns	0.9	1.1	1.0	ns	0.9	1.1	1.1	ns	1.0	1.2
Urban facility (*versus* rural)	1.4	ns	0.6	3.6	0.8	ns	0.4	1.4	0.5	***	0.3	0.7	0.8	ns	0.5	1.3	0.9	ns	0.6	1.1
Provider characteristics																				

For peer review only

For peer review only - http://bmjopen.bmj.com/site/about/guidelines.xhtml

Types (*versus* doctor, advanced pract. clinician)	1.0																			
Nurse, midwife	-				0.7	ns	0.4	1.2	0.6	ns	0.4	1.1	0.9	ns	0.5	1.3	1.1	ns	0.8	1.6
Other - Pharm, lab, dental, non-clinical	-				0.7	ns	0.2	2.5	-				-				0.7	ns	0.4	1.3
Provider is female (*versus* male)	1.8	ns	0.2	20.9	1.0	ns	0.5	2.0	0.8	ns	0.5	1.2	1.2	ns	0.8	1.9	1.0	ns	0.8	1.3
Years since graduation or qualification	1.0	ns	0.9	1.1	1.0	ns	1.0	1.0	1.0	ns	1.0	1.0	1.0	ns	1.0	1.0	1.0	ns	1.0	1.0
Regular salary supplements for provider (*vs* none)	1.2	ns	0.5	3.0	0.7	ns	0.3	1.5	1.3	ns	0.7	2.3	1.0	ns	0.7	1.4	1.1	ns	0.8	1.4
Process of care																				
Technical quality of care observed	0.8	ns	0.6	1.1	1.0	ns	0.8	1.1	1.2	*	1.0	1.4	1.0	ns	0.9	1.1	1.2	***	1.1	1.3
Problem with days or hours services are provided	1.0	ns	0.4	2.7	1.5	ns	0.7	3.3	0.6	ns	0.3	1.1	0.8	ns	0.5	1.2	1.0	ns	0.7	1.5
Problem with wait time	0.5	ns	0.2	1.1	0.5	ns	0.3	1.0	0.3	***	0.2	0.4	0.7	*	0.5	1.0	0.6	***	0.4	0.7
Problem with cleanliness of facility	0.7	ns	0.2	2.2	0.9	ns	0.5	1.9	0.5	*	0.2	1.0	0.7	ns	0.4	1.0	0.6	**	0.4	0.8
Problem with how staff treated patient	0.2	***	0.1	0.5	0.8	ns	0.3	2.1	0.2	***	0.1	0.5	0.4	***	0.2	0.6	0.6	ns	0.4	1.0
Problem with ability to discuss concerns with provider	0.3	**	0.1	0.9	2.2	ns	0.7	6.5	1.6	ns	0.8	2.9	0.6	ns	0.3	1.2	0.6	*	0.4	1.0
Problem with amount of explanation provided	0.2	***	0.1	0.5	0.7	ns	0.3	1.9	0.4	**	0.2	0.8	0.6	ns	0.3	1.2	0.4	***	0.2	0.7
Problem with medication availability	0.4	**	0.2	0.8	0.2	***	0.1	0.3	0.5	**	0.3	0.8	0.5	***	0.3	0.7	0.2	***	0.1	0.2
Problem with cost (vs none)	0.2	***	0.1	0.5	0.5	***	0.3	0.8	0.5	**	0.2	0.9	0.6	*	0.3	1.0	0.5	***	0.3	0.8
Problem with cost not specified (vs none)	-				0.9	ns	0.3	2.9	-				1		0.4	2.4	1.21		0.2	9.2

* p ≤ 0.05; ** p≤ 0.01; *** p≤ 0.001

Numbers were rounded to 1 decimal place for space

For peer review only - http://bmjopen.bmj.com/site/about/guidelines.xhtml

For peer review only

DISCUSSION

Based on combined data from five countries, we found that factors related to the process of care during consultations for children under the age of five explained the highest proportion of variance in caretaker satisfaction (13.8 %), followed by factors related to the health systems' foundations (3.8%). Patient and caretakers' characteristics explained the smallest proportion of variance in caretaker satisfaction (0.9%). The children's number of symptoms and the caregiver's higher education levels both decreased the odds of being very satisfaction. The facility type and the readiness to provide sick child services also predicted overall satisfaction, although the providers' characteristics did not. In terms of process of care, the odds of being very satisfied were lower for caretakers who were not given adequate explanations and those who had encountered problems with medication availability and the cost of services.

To our knowledge, this is the first multi-country study to examine factors affecting variation in caretaker satisfaction with sick child consultations in multiple LMICs. Several findings deserve attention. First and foremost, in both the pooled and country-stratified analyses, user-reported problems with process of care accounted for the largest proportion of variance explained in caretaker satisfaction with sick child care. This finding is consistent with two previous studies. In Peru, Leslie *et al* [20] found that user experience accounted for a larger proportion of variance in all user satisfaction measures, including three and five categories of net promoter score (NPS) and satisfaction level, than the proportion explained by individual, facility, and contextual factors combined. Similarly, Bleich *et al*'s study [4] based on 21 European Union countries reported that all variables considered in the analyses (e.g., user experience, user expectations) accounted for 17.5% of the observed variation in user satisfaction, of which 10% was explained by user experience.

For peer review only - http://bmjopen.bmj.com/site/about/guidelines.xhtml

Second, technical quality of care, a sub-dimension of process of care, was a significant predictor of caretaker satisfaction with the service. Odds of caretakers' being very satisfied with the service increased 1.08 times with every 10% increase in technical quality score—a substantial effect size if quality were 30% or 40% higher, which is conceivable given low baseline performance. However, this association was not significant in three of the five countries. The reason for this may be that, in some contexts, patients find it difficult to assess technical quality of care due to lack of medical expertise [21]. In contrast, it may be easier for patients to perceive tangible aspects, such as outcome quality (e.g., how much the symptoms have been improved) [22,23], and assess their user experience (e.g., wait times, affordability of care) [24–26]. This should be taken into account when user satisfaction is used to assess the performance of facilities.

Third, we found substantial between-country heterogeneity. The proportion of the total variance explained by the variables was notably higher in Afghanistan (51.7 %, as opposed to 9.0%~28.6% in the other four countries). Also, the proportion of variance explained by process of care was highest in Afghanistan (increase in R squared: 36.6% in Afghanistan vs. 5.8%~24.2%). Compared to other countries, the provider's courtesy and communication (e.g., explanations, discussions) appeared to be particularly important aspects of caretaker satisfaction in Afghanistan. In DRC, total variance explained by the variables was only 9%, suggesting there were still more variables influencing caretaker satisfaction, which we were unable to take into account (e.g., insecurity, unpaid government employees). According to Bleich *et al* [4], external factors such as media coverage could influence how satisfied people are with their healthcare system. Future qualitative and mixed methods studies could be useful to explore and uncover new context-specific factors that affect variance in user satisfaction.

Factors predicting patient satisfaction also differed between countries. Experiences of problems with medication availability or cost were the only indicators related to user experience that had a marked negative association with caretaker satisfaction across all five countries. On the other hand, inability to discuss concerns with providers and problems with how staff treated

For peer review only - http://bmjopen.bmj.com/site/about/guidelines.xhtml

patients showed significant negative association with caretaker satisfaction in only a few countries, whereas it was identified as a strong predictor of user satisfaction in earlier studies conducted on different types of maternal care in other countries. Specifically, Dzomeku found that providers' unfriendliness and ineffective communication negatively affected maternal satisfaction in Ghana [27]. Similar results were found in Lebanon and Gambia, where it was reported that women were more satisfied with services when they had the providers' attention and adequate communication with them [28,29]. As such, factors linked to patient satisfaction may depend on context, which is related to various expectations, public sentiment, values, or norms [2,30]. These findings can inform strategic priorities in each country aimed at enhancing patient satisfaction with sick child consultations and possibly patient retention.

This study has several limitations. First, the satisfaction rates reported by caretakers were generally high, possibly due to desirability bias in participants' responses during interviews, low patient expectations, and behaviors during the audits. The observation of the providers' performance during the consultation potentially biased the patient – provider interaction. Second, some potential predictors of caretaker satisfaction, such as patients' and providers' personalities and past experiences with the healthcare system, were not available from the SPA surveys and thus were not included in our analysis. Finally, because this is a cross-sectional analysis, the associations identified cannot be interpreted causally. Despite this limitation, our study has the advantage of combining multiple sources of data (facility audits, health provider interviews, exit interviews with caretakers). It is also the first multi-country analysis to examine the determinants of variation in caretaker satisfaction for sick child care in LMICs.

The study is useful to develop a research agenda. First, in our analyses, there is still a substantial proportion of unexplained variation in caretakers' satisfaction. Future research should explore new determinants of variation in patient satisfaction throughout children's care pathways.

For peer review only - http://bmjopen.bmj.com/site/about/guidelines.xhtml

Second, the heterogeneity between countries supports the need for more studies on how cultural differences affect patient satisfaction.

CONCLUSION

This study showed that the process of care explained the largest proportion of variance in caretaker satisfaction during sick child consultations in five LMICs. Caretakers who encountered problems related to waiting time, how the staff treated them, the amount of explanation provided, medication availability and cost of services were less likely to be very satisfied. High-quality healthcare systems should be able to satisfy users, including caretakers of sick children. People who are satisfied may have more confidence in their healthcare system and be more likely to seek care. In this regard, it is important to understand which factors shape variation in user satisfaction in different settings. Interventions targeting patient experience may be effective to improve patient satisfaction, thereby positively influencing utilization rates and patient retention over the course of care.

ACKNOWLEDGEMENTS

The authors express their gratitude to all actors involved in collecting and reporting Service Provider Assessment data in the participating countries.

For peer review only - http://bmjopen.bmj.com/site/about/guidelines.xhtml

FUNDING STATEMENT

This work was supported by the Bill & Melinda Gates Foundation (grants INV-005254). The first author received a fellowship from the Fonds de recherche du Québec – Santé. These *funding sources had no role in the design of this study* the analyses or interpretation of the data.

AUTHORS' CONTRIBUTIONS

AMTT developed the idea, conducted the analyses and wrote the first draft of the manuscript. HYL validated the results and reviewed the manuscript. MEK helped develop the original idea, provided the database, oversaw the analyses and reviewed the manuscript.

ETHICAL APPROVAL

The original survey implementers obtained ethical approvals for data collection; the Harvard University Human Research Protection Program approved this secondary analysis as exempt from human subjects review.

COMPETING INTERESTS

The authors declare no competing interests.

DATA AVAILABILITY STATEMENT

The Service provider data used for these analyses are publicly available through the Demographic and Health Survey (DHS) Program of United States Agency for International Development (USAID). Before you can download datasets, you must register as a DHS data user on this website: https://dhsprogram.com/data/new-user-registration.cfm. The data for each country can be accessed online via the following websites:

- Afghanistan SPA dataset: https://dhsprogram.com/data/dataset/Afghanistan_SPA_2018.cfm
- Democratic Republic of the Congo (DRC) SPA dataset: https://dhsprogram.com/data/dataset/Congo-Democratic-Republic_SPA_2017.cfm
- Haiti SPA dataset: https://dhsprogram.com/data/dataset/Haiti_SPA_2017.cfm
- Malawi SPA dataset: https://dhsprogram.com/data/dataset/Malawi_SPA_2013.cfm
- Tanzania SPA dataset: https://dhsprogram.com/data/dataset/Tanzania_SPA_2014.cfm

For peer review only - http://bmjopen.bmj.com/site/about/guidelines.xhtml

REFERENCES

1 Kruk ME, Gage AD, Arsenault C, *et al.* High-quality health systems in the Sustainable Development Goals era: time for a revolution. *The Lancet Global Health* 2018;**6**:e1196–252. doi:10.1016/S2214-109X(18)30386-3

2 Batbaatar E, Dorjdagva J, Luvsannyam A, *et al.* Determinants of patient satisfaction: a systematic review. *Perspect Public Health* 2017;**137**:89–101. doi:10.1177/1757913916634136

3 McCormick MC, Escobar GJ, Zheng Z, *et al.* Factors influencing parental satisfaction with neonatal intensive care among the families of moderately premature infants. *Pediatrics* 2008;**121**:1111–8. doi:10.1542/peds.2007-1700

4 Bleich SN, Ozaltin E, Murray CKL. How does satisfaction with the health-care system relate to patient experience? *Bull World Health Organ* 2009;**87**:271–8. doi:10.2471/blt.07.050401

[dataset] 5 Ministry of Public Health/Afghanistan, ICF. Data from Service Provision Assessment (SPA) - Afghanistan. 2018.https://dhsprogram.com/data/dataset/Afghanistan_SPA_2018.cfm

[dataset] 6 Ecole de Santé Publique de l'Université de Kinshasa, ICF. Data from Service Provision Assessment (SPA) - République Démocratique du Congo. 2018.https://dhsprogram.com/data/dataset/Congo-Democratic-Republic_SPA_2017.cfm

[dataset] 7 Institut Haïtien de l'Enfance (IHE), ICF International. Data from Service Provision Assessment (SPA) - Haïti. 2018.https://dhsprogram.com/data/dataset/Haiti_SPA_2017.cfm

[dataset] 8 Ministry of Health (MoH) [Malawi], ICF International. Data set from Service Provision Assessment (SPA) - Malawi. 2014.https://dhsprogram.com/data/dataset/Malawi_SPA_2013.cfm

[dataset] 9 Ministry of Health and Social Welfare (MoHSW) [Tanzania Mainland], Ministry of Health (MoH) [Zanzibar], National Bureau of Statistics, *et al.* Data from Service Provision Assessment (SPA) - Tanzania. 2015.https://dhsprogram.com/data/dataset/Tanzania_SPA_2014.cfm

10 DHS Program. SPA Overview. https://dhsprogram.com/Methodology/Survey-Types/SPA.cfm

11 Institut Haïtien de l'Enfance (IHE), ICF International. Évaluation de la Prestation des Services de Soins de Santé, Haïti, 2017-2018. Rockville, Maryland, USA: : IHE & ICF International 2019.

For peer review only - http://bmjopen.bmj.com/site/about/guidelines.xhtml

12 Ministry of Public Health/Afghanistan, ICF. Afghanistan. Service Provision Assessment Survey 2018-19. Kabul, Afghanistan: : Ministry of Public Health, Afghanistan, and ICF 2019.

13 Ecole de Santé Publique de l'Université de Kinshasa (ESPK), ICF. République Démocratique du Congo. Évaluation des Prestations des Services de soins de Santé (EPSS) 2017-2018. Kinshasa, RDC et Rockville, Maryland, USA: : ESPK ICF 2019.

14 Ministry of Health (MoH) [Malawi], ICF International. Malawi. Service Provision Assessment (SPA) 2013-14. Lilongwe, Malawi, and Rockville, Maryland, USA: : MoH and ICF International 2014.

15 Ministry of Health and Social Welfare (MoHSW) [Tanzania Mainland], Ministry of Health (MoH) [Zanzibar], National Bureau of Statistics, *et al.* Tanzania Service Provision Assessment Survey (TSPA) 2014-15. Dar es Salaam, Tanzania, and Rockville, Maryland, US: : MoHSW, MoH, NBS, OCGS, and ICF International 2015.

16 World Health Organization. Service availability and readiness assessment (SARA): an annual monitoring system for service delivery: reference manual. Geneva: : World Health Organization 2013.

17 World Health Organization. Partnership for maternal and newborn health. Opportunities for Africas newborns: practical data policy and programmatic support for newborn care in Africa. Geneva: : World Health Organization 2006.

18 Gera T, Shah D, Garner P, *et al.* Integrated management of childhood illness (IMCI) strategy for children under five. *Cochrane Database Syst Rev* 2016;**6**. doi:10.1002/14651858.CD010123.pub2

19 Snijders T, Bosker R. *Multilevel analysis. An introduction to basic and advanced multilevel modeling*. 2nd edition. London: : Sage Publications 2012.

20 Leslie HH, Lee H-Y, Blouin B, *et al.* Evaluating patient-reported outcome measures in Peru: a cross-sectional study of satisfaction and net promoter score using the 2016 EnSuSalud survey. *BMJ Qual Saf* 2022;**31**:599–608. doi:10.1136/bmjqs-2021-014095

21 Hanefeld J, Powell-Jackson T, Balabanova D. Understanding and measuring quality of care: dealing with complexity. *Bull World Health Organ* 2017;**95**:368–74. doi:10.2471/BLT.16.179309

22 Schaal T, Schoenfelder T, Klewer J, *et al.* Effects of perceptions of care, medical advice, and hospital quality on patient satisfaction after primary total knee replacement: a cross-sectional study. *PLoS One* 2017;**12**:e0178591. doi:10.1371/journal.pone.0178591

For peer review only

23 Naik J RK, Byram A. Healthcare service quality effect on patient satisfaction and behavioural intentions in corporate hospitals in India. *International Journal of Pharmaceutical and Healthcare Marketing* 2016;**10**:48–74. doi:10.1108/IJPHM-07-2014-0043

24 Srivastava A, Avan BI, Rajbangshi P, *et al.* Determinants of women's satisfaction with maternal health care: a review of literature from developing countries. *BMC Pregnancy Childbirth* 2015;**15**:97. doi:10.1186/s12884-015-0525-0

25 Bazant ES, Koenig MA. Women's satisfaction with delivery care in Nairobi's informal settlements. *Int J Qual Health Care* 2009;**21**:79–86. doi:10.1093/intqhc/mzn058

26 George A. Quality of reproductive care in private hospitals in Andhra Pradesh: women's perception. *Economic and Political Weekly* 2002;**37**:1686–92.

27 Dzomeku V. Maternal satisfaction with care during labour: a case-study of the Mampong-Ashanti District Hospital maternity unit in Ghana. *International Journal of Nursing and Midwifery* 2011;**3**:30–4.

28 Kabakian-Khasholian T, Campbell O, Shediac-Rizkallah M, *et al.* Women's experiences of maternity care: satisfaction or passivity? *Soc Sci Med* 2000;**51**:103–13. doi:10.1016/s0277-9536(99)00443-8

29 Jallow IK, Chou Y-J, Liu T-L, *et al.* Women's perception of antenatal care services in public and private clinics in the Gambia. *Int J Qual Health Care* 2012;**24**:595–600. doi:10.1093/intqhc/mzs033

30 Sanchez-Piedra CA, Jaruseviciene L, Prado-Galbarro FJ, *et al.* Factors associated with professional satisfaction in primary care: esults from EUprimecare project. *Eur J Gen Pract* 2017;**23**:114–20. doi:10.1080/13814788.2017.1305350

For peer review only

For peer review only - http://bmjopen.bmj.com/site/about/guidelines.xhtml

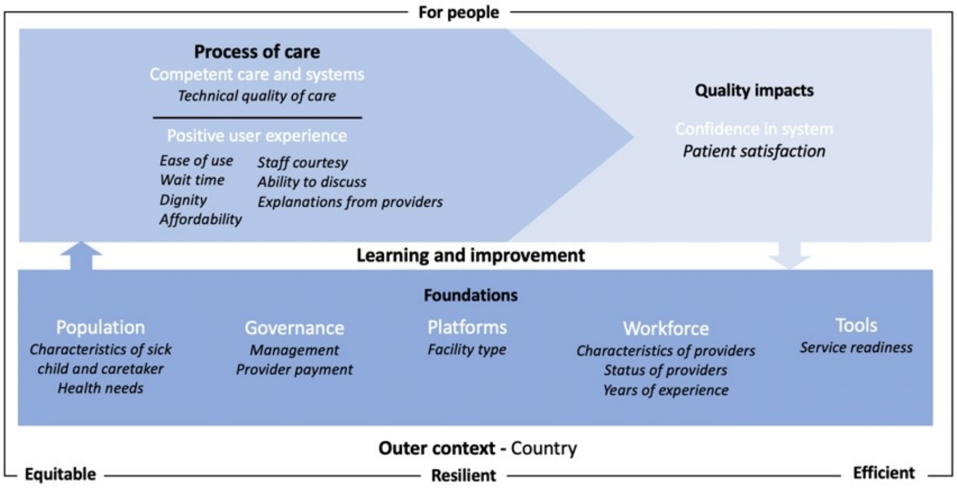

Adapted with permission from Kruk, Gage, Arsenault, et al., 2018

Figure 1. Conceptual framework

156x83mm (144 x 144 DPI)

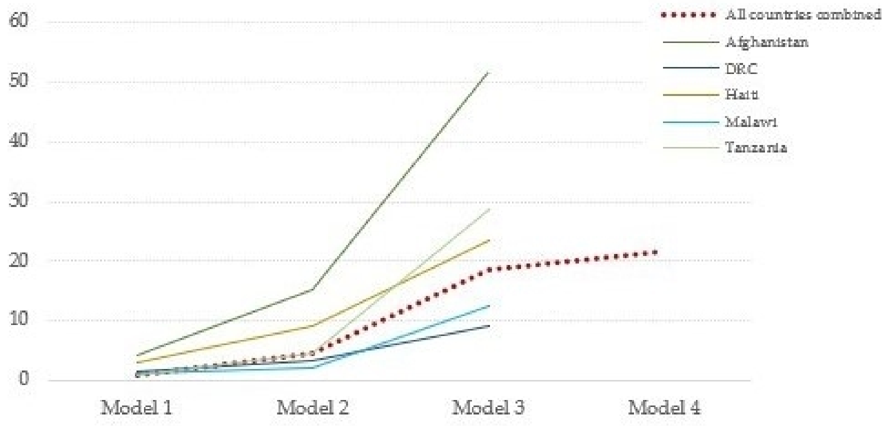

	All countries combined	Afghanistan	DRC	Haiti	Malawi	Tanzania
Null model	0.0	0.0	0.0	0.0	0.0	0.0
Model 1	0.9	4.3	1.5	3.1	1.3	0.8
Model 2	4.7	15.1	3.2	9.2	2.2	4.4
Model 3	18.5	51.7	9.0	23.6	12.6	28.6
Model 4	21.8	NA	NA	NA	NA	NA

Figure 2. Percentage of variance explained with addition of variables to the model

145x122mm (96 x 96 DPI)

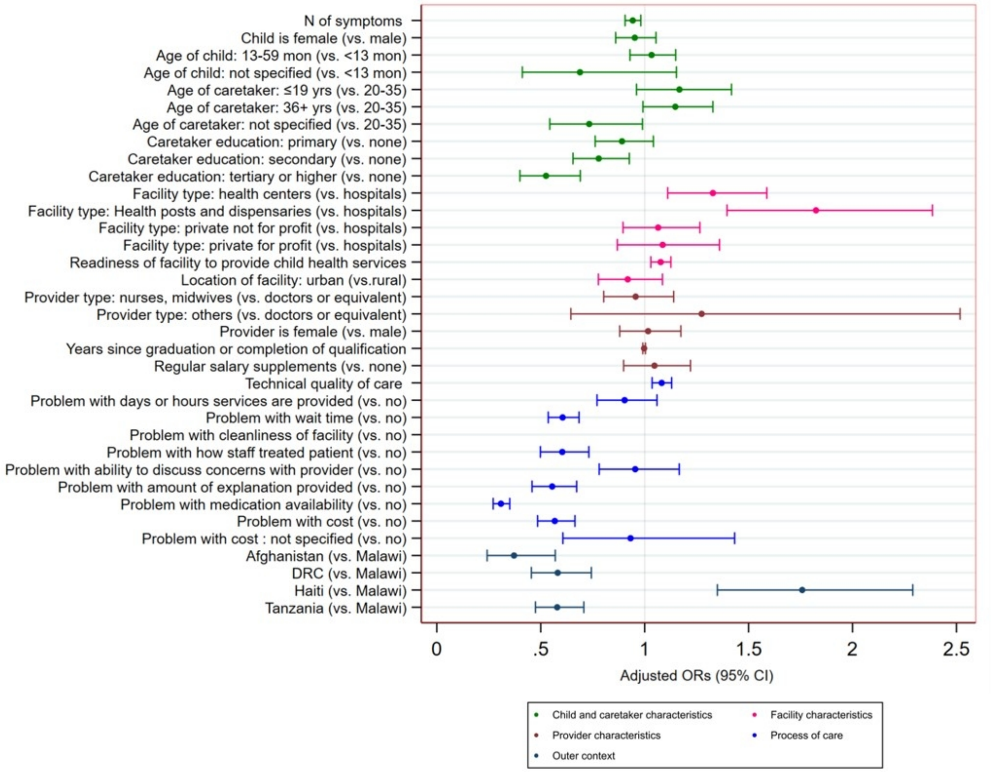

Figure 3. Determinants of caretaker satisfaction

181x143mm (144 x 144 DPI)

Supplementary Files

Box 1. Explained proportion of variance

We applied Snijders and Bosker's method to measure the explained proportion of variance in the logistic multilevel model. We assumed that the dichotomous outcome Y could be conceived as generated through a threshold model with underlying variable $\breve{Y}$, denoted as:

$$\breve{Y}_{ij} = \gamma^0 + \sum_{h=1}^{\gamma} \gamma^h X_{hji} + U_{0j} + R_{ij}$$

Hence the total variance of Y_{ij} was equal to:

$$\text{Var}\,(Y_{ij}) = \sigma_F^2 + \tau_0^2 + \sigma_R^2$$

where σ_F^2 is the explained variance, which is the observed variance of the linear predictor $\hat{Y}_{ij} = \gamma^0 + \sum_{h=1}^{\gamma} \gamma^h X_{hji}$, and τ_0^2 and σ_R^2 are unexplained variances at levels 2 and 1, which were var (U_{0j}) and var (R_{ij}), respectively. The proportion of total variance explained by the fixed effects in the model, corresponding to an R^2 equivalent for multilevel logistic regression, is defined by:

$$R_{dicho}^2 = \frac{\sigma_F^2}{\sigma_F^2 + \tau_0^2 + \sigma_R^2}$$

When considering only the unexplained part of the total variance, the proportion attributed to the health facilities level (called residual intraclass correlation) was evaluated as:

$$\rho_I = \frac{\tau_0^2}{\tau_0^2 + \sigma_R^2}$$

For peer review only

Table S1. Items included in the service readiness indicator for preventive and curative child health services

	Afghanistan (N = 556)	DRC (N = 2592)	Haiti (N = 2093)	Malawi (N = 3177)	Tanzania (N = 4731)	Total (N = 13149)
Staff and training						
Guidelines for IMCI	90 (16.2%)	1408 (54.3%)	483 (23.1%)	1364 (42.9%)	3006 (63.5%)	6351 (48.3%)
Guidelines for growth monitoring	86 (27.5%)	1057 (58.9%)	599 (44.9%)	1089 (38.5%)	1444 (32.8%)	4275 (40.1%)
Staff trained in IMCI	113 (20.3%)	1575 (60.8%)	716 (34.2%)	1041 (32.8%)	1430 (30.2%)	4875 (37.1%)
Equipment						
Child and infant scale	505 (90.8%)	2347 (90.5%)	1775 (84.8%)	2714 (85.4%)	3085 (65.2%)	10426 (79.3%)
Length/height measuring equipment	154 (49.2%)	1274 (70.9%)	1162 (87.2%)	2546 (90.0%)	1865 (42.4%)	7001 (65.6%)
Thermometer	420 (75.5%)	2482 (95.8%)	1975 (94.4%)	2741 (86.3%)	3991 (84.4%)	11609 (88.3%)
Stethoscope	551 (99.1%)	2426 (93.6%)	2051 (98.0%)	2932 (92.3%)	4374 (92.5%)	12334 (93.8%)
Growth chart	123 (39.3%)	1592 (88.6%)	691 (51.8%)	1785 (63.1%)	3191 (72.5%)	7382 (69.2%)
Diagnostics						
Haemoglobin / anemia testing	389 (70.0%)	2039 (83.3%)	332 (19.9%)	806 (28.6%)	2348 (65.1%)	5914 (53.3%)
Test for parasite in stool (microscopy)	446 (80.2%)	1895 (77.4%)	1456 (87.1%)	773 (27.5%)	2236 (62.0%)	6806 (61.3%)
Malaria testing	464 (83.5%)	2316 (94.6%)	1600 (95.7%)	2522 (89.6%)	3286 (91.1%)	10188 (91.8%)
Medicines and commodities						
Oral rehydration	496 (89.2%)	2092 (81.5%)	1427 (68.2%)	2899 (91.5%)	4021 (85.2%)	10935 (83.4%)
Amoxicillin syrup or tablets	528 (95.0%)	2167 (84.4%)	1973 (94.3%)	2949 (93.1%)	4197 (88.9%)	11814 (90.2%)
Co-trimoxazole syrup or suspension	442 (79.5%)	1339 (52.2%)	1560 (74.5%)	2379 (75.1%)	3683 (78.0%)	9403 (71.8%)
Paracetamol syrup or suspension	493 (88.7%)	1188 (46.3%)	1720 (82.2%)	2304 (72.7%)	3134 (66.4%)	8839 (67.5%)
Vitamin A capsule	470 (84.5%)	1170 (45.6%)	1258 (60.1%)	1477 (46.6%)	3119 (66.1%)	7494 (57.2%)
Albendazole or mebendazole	488 (87.8%)	2174 (84.7%)	1861 (88.9%)	3062 (96.7%)	4227 (89.6%)	11812 (90.1%)
Zinc tablet or syrup	409 (73.6%)	1729 (67.4%)	919 (43.9%)	2722 (85.9%)	2578 (54.6%)	8357 (63.8%)

For peer review only

Table S2. Items included in the technical quality indicator for children older than 2 months

	**Afghanistan** (N = 516)	**DRC** (N = 2452)	**Haiti** (N = 1799)	**Malawi** (N = 3029)	**Tanzania** (N = 4516)	**Total** (N = 12312)
Provider asked / caretaker mentioned if child unable to drink or breastfeed	70 (13.8%)	526 (21.0%)	319 (18.1%)	848 (27.7%)	1348 (29.7%)	3111 (25.1%)
Provider asked / caretaker mentioned cough or difficult breathing	337 (66.5%)	1576 (63.0%)	1223 (69.4%)	2206 (72.0%)	3498 (77.0%)	8840 (71.4%)
Provider asked / caretaker mentioned diarrhea	203 (40.0%)	1105 (44.1%)	648 (36.8%)	1273 (41.5%)	2813 (62.0%)	6042 (48.8%)
Provider asked / caretaker mentioned fever	384 (75.7%)	2251 (89.9%)	1274 (72.3%)	2465 (80.4%)	4216 (92.9%)	10590 (85.6%)
Provider asked / caretaker mentioned vomiting	180 (35.5%)	1084 (43.3%)	486 (27.6%)	1134 (37.0%)	2352 (51.8%)	5236 (42.3%)
Provider asked / caretaker mentioned convulsions	32 (6.3%)	259 (10.3%)	69 (3.9%)	285 (9.3%)	1041 (22.9%)	1686 (13.6%)
Provider asked about mother's HIV status	0 (0.0%)	36 (1.4%)	36 (2.0%)	216 (7.0%)	377 (8.3%)	665 (5.4%)
Provider asked / caretaker mentioned ear pain	54 (10.7%)	130 (5.2%)	102 (5.8%)	161 (5.3%)	767 (16.9%)	1214 (9.8%)
Provider counted respiration for 60 seconds	79 (15.6%)	691 (27.6%)	366 (20.8%)	501 (16.3%)	644 (14.2%)	2281 (18.4%)
Provider weighed client	240 (47.3%)	1903 (76.0%)	1446 (82.1%)	638 (20.8%)	673 (14.8%)	4900 (39.6%)
Provider plotted weight on growth chart	28 (5.5%)	154 (6.2%)	178 (10.1%)	179 (5.8%)	190 (4.2%)	729 (5.9%)
Provider took temperature	308 (60.7%)	2334 (93.2%)	1562 (88.7%)	2131 (69.5%)	2801 (61.7%)	9136 (73.8%)
Provider checked palms / conjunctiva / mouth for pallor	112 (22.1%)	1911 (76.3%)	909 (51.6%)	1429 (46.6%)	2052 (45.2%)	6413 (51.8%)
Provider checked for oedema	27 (5.3%)	235 (9.4%)	101 (5.7%)	266 (8.7%)	334 (7.4%)	963 (7.8%)
Provider checked vaccination card or vaccinated	54 (10.7%)	551 (22.0%)	475 (27.0%)	2412 (78.7%)	1716 (37.8%)	5208 (42.1%)
Provider asked if child received vitamin A within past 6months	4 (0.8%)	146 (5.8%)	41 (2.3%)	71 (2.3%)	174 (3.8%)	436 (3.5%)
Provider asked if child received any deworming medication in past 6 months	5 (1.0%)	303 (12.1%)	54 (3.1%)	69 (2.3%)	266 (5.9%)	697 (5.6%)
Provider explained dosing if medication prescribed	225 (45.6%)	1220 (49.9%)	1043 (65.8%)	1597 (52.6%)	1686 (38.0%)	5771 (48.1%)
Provider recommended food / liquid intake	174 (34.3%)	680 (27.2%)	694 (39.4%)	581 (18.9%)	1190 (26.2%)	3319 (26.8%)
Provider described ≥ 1 danger sign requiring return to facility	44 (8.7%)	289 (11.5%)	90 (5.1%)	379 (12.4%)	770 (17.0%)	1572 (12.7%)
Provider discussed follow-up appointment	26 (5.1%)	340 (13.6%)	628 (35.7%)	744 (24.3%)	1016 (22.4%)	2754 (22.3%)
Provider stated diagnosis to caretaker	178 (35.1%)	846 (33.8%)	266 (15.1%)	1365 (44.5%)	2205 (48.6%)	4860 (39.3%)

For peer review only

Table S3. Items included in the technical quality indicator for children 2 months or younger

	Afghanistan	DRC	Haiti	Malawi	Tanzania	Total
	(N = 49)	(N = 87)	(N = 341)	(N = 109)	(N = 191)	(N = 777)
Provider asked / caretaker mentioned if child unable to drink or breastfeed	15 (30.6%)	39 (43.8%)	97 (29.2%)	52 (46.8%)	74 (38.7%)	277 (35.9%)
Provider asked about normal (breast)feeding pattern	20 (40.8%)	38 (42.7%)	165 (49.7%)	21 (18.9%)	81 (42.4%)	325 (42.1%)
Provider asked about (breast)feeding pattern during this illness	12 (24.5%)	24 (27.0%)	104 (31.3%)	25 (22.5%)	53 (27.7%)	218 (28.2%)
Provider asked / caretaker mentioned diarrhea	19 (38.8%)	24 (27.0%)	49 (14.8%)	28 (25.2%)	89 (46.6%)	209 (27.1%)
Provider asked / caretaker mentioned convulsions	4 (8.2%)	10 (11.2%)	8 (2.4%)	7 (6.3%)	40 (20.9%)	69 (8.9%)
Provider asked about mother's HIV status	0 (0.0%)	1 (1.1%)	17 (5.1%)	9 (8.1%)	24 (12.6%)	51 (6.6%)
Provider counted respiration for 60 seconds	8 (16.3%)	37 (41.6%)	82 (24.7%)	20 (18.0%)	29 (15.2%)	176 (22.8%)
Provider weighed client	35 (71.4%)	66 (74.2%)	273 (82.2%)	24 (21.6%)	33 (17.3%)	431 (55.8%)
Provider plotted weight on growth chart	2 (4.1%)	3 (3.4%)	39 (11.7%)	4 (3.6%)	12 (6.3%)	60 (7.8%)
Provider took temperature	33 (67.3%)	75 (84.3%)	280 (84.3%)	81 (73.0%)	116 (60.7%)	585 (75.8%)
Provider looked into child's mouth	25 (51.0%)	13 (14.6%)	41 (12.3%)	10 (9.0%)	29 (15.2%)	118 (15.3%)
Provider checked vaccination card or vaccinated	8 (16.3%)	32 (36.0%)	124 (37.3%)	97 (87.4%)	86 (45.0%)	347 (44.9%)
Provider asked if child received vitamin A within past 6 months	0 (0.0%)	4 (4.5%)	3 (0.9%)	2 (1.8%)	3 (1.6%)	12 (1.6%)
Provider explained dosing if medication prescribed	21 (53.8%)	43 (51.2%)	165 (59.4%)	53 (50.0%)	48 (27.3%)	330 (48.3%)
Provider recommended food / liquid intake	27 (55.1%)	31 (34.8%)	190 (57.2%)	48 (43.2%)	55 (28.8%)	351 (45.5%)
Provider described ≥ 1 danger sign requiring return to facility	6 (12.2%)	9 (10.1%)	23 (6.9%)	20 (18.0%)	33 (17.3%)	91 (11.8%)
Provider discussed follow-up appt (sick child)	3(6.1%)	15 (16.9%)	115 (34.6%)	24 (21.6%)	44 (23.0%)	201 (26.0%)

For peer review only

Table S4. Modeling strategy for analyses

Model #	Description	Variables included
Model 0 A	Empty model no random intercept	
Model 0 B	Empty model with random intercept	
Model 1	Child and caretaker characteristics	Number of symptoms caretaker identified<br>Child gender<br>Age of child<br>Age of caretaker<br>Education level of caretaker
Model 2	Model 1 + health system foundations	Facility type<br>Facility management<br>Readiness of facility to provide child health services<br>Urban setting<br>Provider type<br>Provider gender<br>Provider years since graduation<br>Provider receives regular salary supplement
Model 3	Model 2 + Process of care	Technical quality of care observed<br>Problem with days or hours services are provided<br>Problem with wait time<br>Problem with cleanliness of facility<br>Problem with how staff treated patient<br>Problem with ability to discuss concerns with provider<br>Problem with amount of explanation provided<br>Problem with medication<br>Problem with cost of services
Model 4	Model 3 + Outer context	Countries

For peer review only

STROBE Statement—Checklist of items that should be included in reports of ***cross-sectional studies***

	Item No	Recommendation	Page No
**Title and abstract**	1	(*a*) Indicate the study's design with a commonly used term in the title or the abstract	1
		(*b*) Provide in the abstract an informative and balanced summary of what was done and what was found	1
**Introduction**			
Background/rationale	2	Explain the scientific background and rationale for the investigation being reported	3-4
Objectives	3	State specific objectives, including any prespecified hypotheses	4
**Methods**			
Study design	4	Present key elements of study design early in the paper	4
Setting	5	Describe the setting, locations, and relevant dates, including periods of recruitment, exposure, follow-up, and data collection	4
Participants	6	(*a*) Give the eligibility criteria, and the sources and methods of selection of participants	4-5
Variables	7	Clearly define all outcomes, exposures, predictors, potential confounders, and effect modifiers. Give diagnostic criteria, if applicable	5-7
Data sources/ measurement	8*	For each variable of interest, give sources of data and details of methods of assessment (measurement). Describe comparability of assessment methods if there is more than one group	5-7
Bias	9	Describe any efforts to address potential sources of bias	7
Study size	10	Explain how the study size was arrived at	4
Quantitative variables	11	Explain how quantitative variables were handled in the analyses. If applicable, describe which groupings were chosen and why	7
Statistical methods	12	(*a*) Describe all statistical methods, including those used to control for confounding	7
		(*b*) Describe any methods used to examine subgroups and interactions	7
		(*c*) Explain how missing data were addressed	9
		(*d*) If applicable, describe analytical methods taking account of sampling strategy	7
		(*e*) Describe any sensitivity analyses	N/A
**Results**			
Participants	13*	(a) Report numbers of individuals at each stage of study—eg numbers potentially eligible, examined for eligibility, confirmed eligible, included in the study, completing follow-up, and analysed	9
		(b) Give reasons for non-participation at each stage	N/A
		(c) Consider use of a flow diagram	N/A

For peer review only

Descriptive data	14*	(a) Give characteristics of study participants (eg demographic, clinical, social) and information on exposures and potential confounders	9-10
		(b) Indicate number of participants with missing data for each variable of interest	9
Outcome data	15*	Report numbers of outcome events or summary measures	Table 2, page 9-10
Main results	16	(*a*) Give unadjusted estimates and, if applicable, confounder-adjusted estimates and their precision (eg, 95% confidence interval). Make clear which confounders were adjusted for and why they were included	10-12
		(*b*) Report category boundaries when continuous variables were categorized	5-6
		(*c*) If relevant, consider translating estimates of relative risk into absolute risk for a meaningful time period	N/A
Other analyses	17	Report other analyses done—eg analyses of subgroups and interactions, and sensitivity analyses	13-14 and supplementary file
**Discussion**			
Key results	18	Summarise key results with reference to study objectives	15
Limitations	19	Discuss limitations of the study, taking into account sources of potential bias or imprecision. Discuss both direction and magnitude of any potential bias	16-17
Interpretation	20	Give a cautious overall interpretation of results considering objectives, limitations, multiplicity of analyses, results from similar studies, and other relevant evidence	15-17
Generalisability	21	Discuss the generalisability (external validity) of the study results	16
**Other information**			
Funding	22	Give the source of funding and the role of the funders for the present study and, if applicable, for the original study on which the present article is based	See cover page

*Give information separately for exposed and unexposed groups.

Note: An Explanation and Elaboration article discusses each checklist item and gives methodological background and published examples of transparent reporting. The STROBE checklist is best used in conjunction with this article (freely available on the Web sites of PLoS Medicine at http://www.plosmedicine.org/, Annals of Internal Medicine at http://www.annals.org/, and Epidemiology at http://www.epidem.com/). Information on the STROBE Initiative is available at www.strobe-statement.org.

For peer review only

For peer review only - http://bmjopen.bmj.com/site/about/guidelines.xhtml
